# Lipid signature of advanced human carotid atherosclerosis assessed by mass spectrometry imaging

**DOI:** 10.1194/jlr.RA120000974

**Published:** 2021-01-06

**Authors:** Astrid M. Moerman, Mirjam Visscher, Nuria Slijkhuis, Kim Van Gaalen, Bram Heijs, Theo Klein, Peter C. Burgers, Yolanda B. De Rijke, Heleen M.M. Van Beusekom, Theo M. Luider, Hence J.M. Verhagen, Antonius F.W. Van der Steen, Frank J.H. Gijsen, Kim Van der Heiden, Gijs Van Soest

**Affiliations:** 1 Department of Cardiology, Erasmus MC University Medical Center Rotterdam, Rotterdam, The Netherlands; 2 Center for Proteomics and Metabolomics, Leiden University Medical Center, Leiden, The Netherlands; 3 Department of Clinical Chemistry, Erasmus MC University Medical Center Rotterdam, Rotterdam, The Netherlands; 4 Department of Neurology, Laboratory of Neuro-Oncology, Erasmus MC University Medical Center Rotterdam, Rotterdam, The Netherlands; 5 Department of Experimental Cardiology, Erasmus MC University Medical Center Rotterdam, Rotterdam, The Netherlands; 6 Department of Vascular and Endovascular Surgery, Erasmus MC University Medical Center Rotterdam, Rotterdam, The Netherlands; 7 Shenzhen Institutes of Advanced Technology, Chinese Academy of Sciences, Shenzhen, China

**Keywords:** atherosclerosis, mass spectrometry, lipidomics, lipids, sphingolipids, Vascular Biology, oxidized lipids, imaging, histology, ischemic stroke, sphingomyelin, CE, cholesteryl ester, CEA, carotid endarterectomy, DCM, dichloromethane, DG, diacylglycerol, FC, foam cells, LPC, lysophosphatidylcholine, MSI, mass spectrometry imaging, NC, necrotic core, NMF, nonnegative matrix factorization, OPLS-DA, orthogonal projections to latent structures discriminant analysis, PC, phosphatidylcholine, SM, sphingomyelin, TG, triacylglycerol, VIP, variable influence on projection

## Abstract

Carotid atherosclerosis is a risk factor for ischemic stroke, one of the main causes of mortality and disability worldwide. The disease is characterized by plaques, heterogeneous deposits of lipids, and necrotic debris in the vascular wall, which grow gradually and may remain asymptomatic for decades. However, at some point a plaque can evolve to a high-risk plaque phenotype, which may trigger a cerebrovascular event. Lipids play a key role in the development and progression of atherosclerosis, but the nature of their involvement is not fully understood. Using matrix-assisted laser desorption/ionization mass spectrometry imaging, we visualized the distribution of approximately 200 different lipid signals, originating of >90 uniquely assigned species, in 106 tissue sections of 12 human carotid atherosclerotic plaques. We performed unsupervised classification of the mass spectrometry dataset, as well as a histology-directed multivariate analysis. These data allowed us to extract the spatial lipid patterns associated with morphological plaque features in advanced plaques from a symptomatic population, revealing spatial lipid patterns in atherosclerosis and their relation to histological tissue type. The abundances of sphingomyelin and oxidized cholesteryl ester species were elevated specifically in necrotic intima areas, whereas diacylglycerols and triacylglycerols were spatially correlated to areas containing the coagulation protein fibrin. These results demonstrate a clear colocalization between plaque features and specific lipid classes, as well as individual lipid species in high-risk atherosclerotic plaques.

Atherosclerosis is a disease causing plaque formation in the inner arterial walls and remains the largest cause of death worldwide (1). The main driving mechanism for atherosclerotic plaque initiation is the gradual accumulation of lipids, originating from circulating lipoproteins, at sites of endothelial dysfunction in the vessel wall (2). The influx of lipids and their subsequent modification in the vessel wall triggers inflammatory reactions that exacerbate the atherogenic process (3, 4). With disease progression, lipids start to play a dual role: they are deposited as metabolites of inflammatory processes and also form lipoproteins that act as signaling molecules, binding to a variety of macrophage-borne receptors (5). Advanced plaques are complex heterogeneous structures that eventually can destabilize or erode to form a nidus for thrombogenesis, triggering ischemic vascular syndrome (6). Previous studies have found that the lipid content of atherosclerotic plaques is different at various stages of disease progression (7, 8), but the detailed interaction between the local vessel wall pathology and lipid biology has not been resolved. Insight into this interaction and its relation with disease stage may be gained by visualizing the molecular lipid composition of plaques (9).

Matrix-assisted laser desorption/ionization mass spectrometry imaging (MALDI-MSI) is a label-free molecular imaging technique and is suitable for detection and visualization of lipids in tissue sections (10, 11). MSI studies of lipid distribution in human atherosclerotic plaques that have been performed to date reported only small numbers of samples or a limited spectrum of lipid species (12, 13, 14, 15, 16, 17, 18). We use a previously established MALDI-MSI pipeline for systematic imaging of lipids (19) to visualize the spatial distribution of 194 lipids in 106 tissue sections of 12 carotid atherosclerotic plaques, harvested at carotid endarterectomy surgeries in 12 patients. Analysis of this dataset identifies spatial lipid patterns in advanced atherosclerosis that transcend individual variability.

We processed tissue sections adjacent to those studied by MSI for a series of histological staining procedures to identify compositional features of plaque vulnerability, i.e., necrotic core, the thrombus-associated protein fibrin, erythrocytes, and foam cells (20, 21, 22). In this way we were able to compare spatial lipid patterns with gold standard histological assessment of plaque composition. In this paper, we describe the systematic analysis of the high-dimensional MALDI-MSI dataset. We assessed spatial correlations between lipids and performed unsupervised cluster analysis. Second, we considered the histological results and investigated correlations between lipids and compositional features of plaque vulnerability.

In this study, we systematically visualize the molecular lipid content of 12 human atherosclerotic plaques with high resolution, and contextualize it in terms of gold standard histological plaque assessments, to provide insight toward the lipid signature of specific plaque features related to plaque vulnerability, such as necrotic core and fibrin.

## Materials and methods

### Tissue collection and processing

Twelve human carotid endarterectomy (CEA) plaque specimens were surgically excised, snap frozen, and stored at −80°C until further processing. The surgery was performed using a protocol that preserves an intact lumen and plaque morphology (23). Upon processing, CEA specimens were divided in 2-mm-thick cross-sections. Each cross-section was embedded in 10% porcine type A gelatin (Sigma-Aldrich, The Netherlands) and cryosectioned (CM3050 S, Leica Biosystems) into 10-μm-thick sections. Tissue sections were thaw-mounted on glass slides and stored at −80°C. One slide was processed for MALDI-MSI, six other slides were histochemically stained.

### Ethics statement

This study was performed according to the ethical guidelines sanctioned by the Ethics Board of Erasmus MC (MEC 2008-147).

### MALDI-MSI sample preparation, measurement, and data reduction

MALDI-MSI experiments and data processing were performed according to the methods described in Visscher *et al*. (19). In short, we desiccated the tissue sections and applied 2,5-dihydroxybenzoic acid matrix by sublimation (home-built sublimation system as described in Dekker *et al*. (24)). MALDI-MSI experiments were performed on a Synapt G2Si-TOF system (Waters, Manchester, United Kingdom), operated in positive ion mode at the instrument's resolution mode (single-pass reflectron TOF, mass resolution of 20,000), using a 2,000 Hz Nd:YAG (355 nm) laser with a pixel-size of 45 × 45 μm^2^ and using Waters Research Enabled Software suite. The mass range was 300–1,200 *m/z*, and the laser fired 100 shots per pixel. The data were acquired using MassLynx v4.2 software, and HDI v1.4 (Waters, Manchester, United Kingdom) was used to export the MSI data in an imzML format and an in-house developed data processing pipeline in MATLAB™ 2017a (Natick, MA) and mMass software (25) was used to select tissue-specific lipid *m/z* values. We selected the lipid *m/z* values that were measured in more than 30% of all tissue sections and removed isotopes from the dataset, different adducts of the same molecule were retained. The data were log transformed for statistical analysis to comply with the assumptions of these methods (26). All figures in this paper show Synapt G2Si-TOF data.

### Spatial cross-correlation

Per tissue section, the spatial correlation of a selection of 70 lipid images was calculated using the Pearson correlation coefficient: ${\text{R}}^{2}=\frac{\sum \left(\left({\text{x}}_{\text{i}}-\bar{\text{x}}\right)\left(\text{y}-\bar{\text{y}}\right)\right)}{{\text{σ}}_{\text{x}}{\text{σ}}_{\text{y}}}$. The results of all tissue sections were averaged to obtain the average Pearson correlation coefficients for our dataset.

### Unsupervised clustering algorithm

We calculated the optimal number of spectral components for this dataset and applied nonnegative matrix factorization (NMF) dimension reduction to the combined spectral data of all tissue sections, using an NMF toolbox for biological datamining (27, 28, 29). We determined the optimum number of components based on dispersion coefficients (30).

### Histology, histology segmentation, and image registration

The tissue section used in the MALDI-MSI experiment was stained for lipids by Oil Red O. Adjacent tissue sections were histochemically stained by Miller's elastic stain, Martius scarlet blue trichrome, and hematoxylin-eosin. Based on the histological information, a segmentation image of the tissue section was drawn, in which plaque components were identified: necrotic core (NC), fibrin, foam cells (FCs), erythrocytes, and calcium. The segmentation images were registered to the MALDI tissue section by translation and scaling using an in-house-developed point-based rigid image registration framework in MeVisLab (MeVis Medical Solutions AG, Germany), to enable correlation of histology and MALDI-MSI data. After registration, the mean spectrum for each plaque component was calculated per tissue section.

### Multivariate analysis

To investigate the presence of lipids characteristic for any plaque component, we performed multivariate analysis. For all plaque components, we fitted an Orthogonal Projections to Latent Structures Discriminant Analysis (OPLS-DA) model (31) in SIMCA 15 (Umetrics, Sweden) comparing the mean spectra of the plaque component with the mean spectra of the rest of the tissue. In these models the intensities of the *m/z* values in the mean spectrum were defined as variables and the histological components were defined as the observations. Quality of fit and predictability of the model were reported by *R*^2^ and Q^2^ values, respectively. The significance of OPLS-DA models was checked by sevenfold cross-validation analysis of variance (32), and the models were validated by permutation tests. If the model was significant, we extracted the variable influence on projection (VIP) values >1.0, which were considered to be present in significantly different amounts (33).

### Comparison of unsupervised NMF clustering with histology-based multivariate analysis

Per NMF component spectrum we calculated a cutoff value of 0.4 times the normalized weight of that NMF component. We identified the *m/z* values with intensities above this cutoff value. We compared these *m/z* values with the *m/z* values with VIP > 1.0 in the multivariate analyses to check the correspondence between the NMF unsupervised clustering and the histology-based multivariate analysis.

### Statistics

The VIP values > 1.0 of the significant OPLS-DA models were separately tested on all data, including the patients for whom the MVA model was not significant. The Wilcoxon signed-rank test was used to statistically test the non-log fold changes in intensity between the segmented and all other tissue areas. *M/z* values that had a *P*-value ≤ 0.05 were considered statistically significant. The receiver operating characteristic was used to determine the optimal cutoff intensity (maximum Youden's index) for the VIP *m/z* values.

### Lipid identification

We performed lipid identification experiments in two ways. First, we performed a lipidomics analysis on homogenized carotid endarterectomy tissue using the Lipidyzer platform (Sciex, Framingham, MA). Nine pieces of 50 mg CEA tissue was homogenized in 2 mL ice-cold methanol/MilliQ water [50/50% (v/v)] with three 10 s bursts using an ultraturrax homogenizer. Homogenates were transferred to 2 mL Eppendorf tubes and stored on ice until centrifugation at 20,000 *g*. The supernatant was collected and submitted to lipidomics analysis. Fifty microliters of tissue homogenate was mixed with the software precalculated amount of isotopically labeled internal standard compounds in 13 lipid classes before liquid-liquid extraction with dichloromethane (DCM)/methanol. After 30 min, the lower phase was collected and the sample re-extracted with DCM. The combined extracts were dried under flowing N_2_ and resuspended in running buffer [10 mM ammonium acetate in DCM/methanol 50/50 (% v/v)]. The extracted samples were analyzed using the manufacturer-provided Lipidyzer SRM methods on a Shimadzu Nexera 20 UPLC system hyphenated to a Sciex 5500 Q-TRAP mass spectrometer equipped with a Selexion differential ion mobility module run with 1-propanol as a modifier. The SRM scan transitions can be found in supplemental Table S1. After analysis, lipid species quantification was performed using the Lipidyzer software, see supplemental Table S2.

In addition, we performed MALDI-FTICR-MSI on a 12 T Bruker Daltonics solariX xR mass spectrometer equipped with dynamically harmonized ParaCell™, and a CombiSource™ (Bruker Daltonics, Bremen, Germany), on a subset of sections. The instrument was operated using ftmsControl (v2.1.0 Build 98; Bruker Daltonics), and data were collected using a transient length of 0.4194 s (512k data point time domain), resulting in an estimated resolution of 97,000 at *m/z* 400. MALDI-measurements were performed using the SmartBeam™-II laser (λ = 355 nm) operating at 200 Hz, at 21% power, and using the “Small” focusing setting (ablation area approximately 70 × 70 μm^2^). A total of 50 shots were acquired per pixel, with a mass range covering 295–1,500 *m/z*. MSI analyses were performed at 70 × 70 μm^2^ and 100 × 100 μm^2^ pixel size.

MSI analysis was performed on one section using a transient length of 3.3554s (4M data point time domain) resulting in an estimated resolution of 776,000 at *m/z* 400, in order to resolve isobaric species. MALDI-measurements were performed using the SmartBeam™-II laser (λ = 355 nm) operating at 200 Hz, at 15% power, and using the “Small” focusing setting (ablation area approximately 70 × 70 μm^2^). A total of 50 shots were acquired per pixel, with a mass range covering 300–1,500 *m/z*. MSI analyses were performed at 100 × 100 μm^2^ pixel size.

Acquired data were loaded in SCiLS Lab (v2016b; Bruker Daltonics) and converted to imzML before further processing. We uploaded the FTICR data to the METASPACE annotation platform (34) and searched the available annotation databases with a false discovery rate < 10%.

## Results

### MALDI-MSI of plaque lipids shows lipid class-specific spatial patterns

**Fig. 1:**
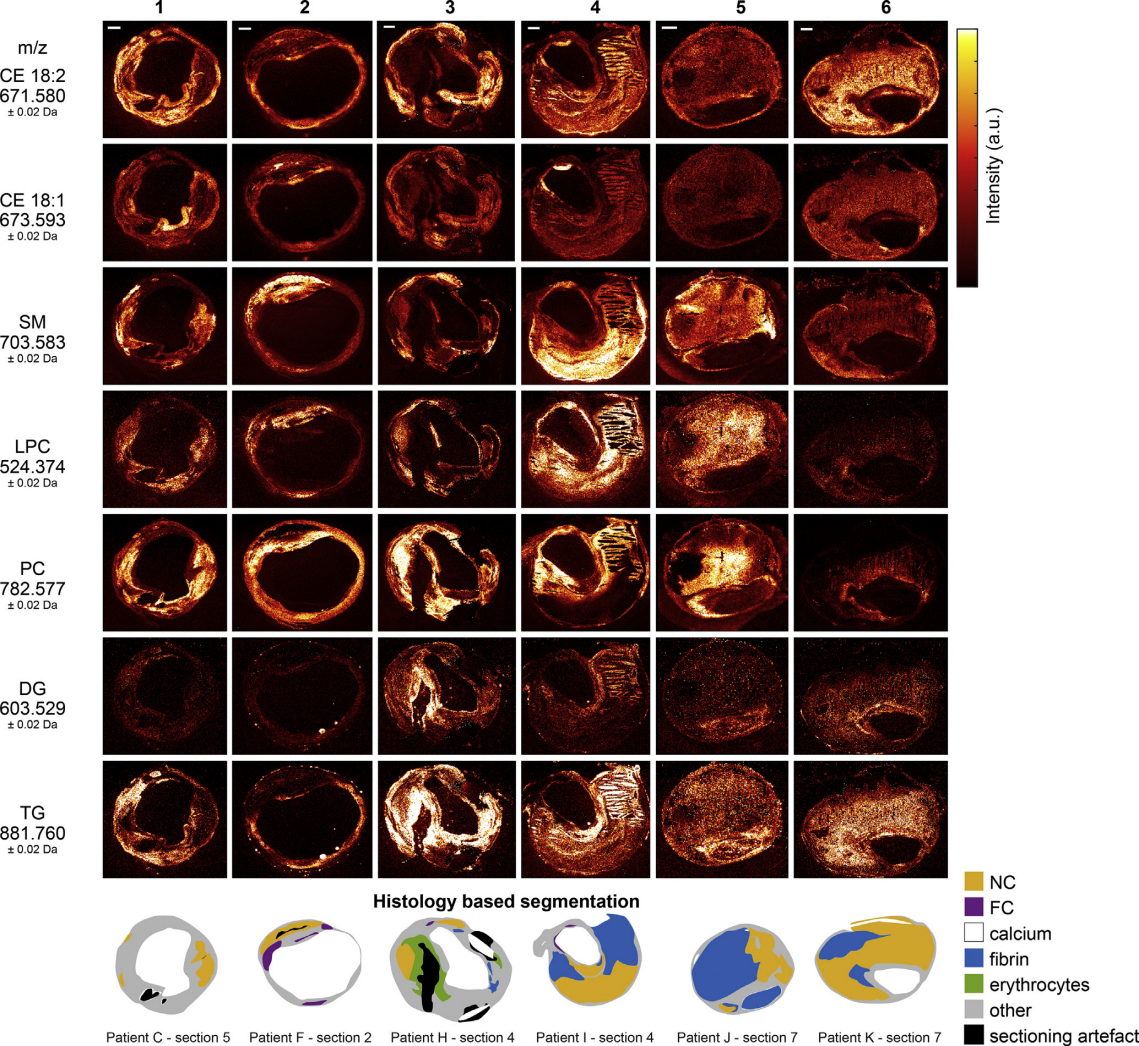
Overview of MALDI-MSI data for a selection of 7 lipids. Data are shown for 6 tissue sections representative of the 106 section dataset, in terms of histological plaque composition. For comparison, histology segmentation images are also shown. The lipid images were normalized per *m/z* value, to enable reliable comparison of the distribution of a specific lipid in different tissue sections. Also, CE(18:2) and CE(18:1) are shown on the same intensity scale, as are DG and TG. Scale bars are 1 mm. CE, cholesterol ester; DG, diacylglycerol; FC, foam cells; LPC, lysophosphatidylcholines; NC, necrotic core; PC, phosphatidylcholines; SM, sphingomyelin; TG, triacylglycerol.

Twelve human carotid endarterectomy samples were processed into a series of 106 axial tissue sections to be imaged by MALDI-MSI and histology. Processing of MALDI-MSI data resulted in lipid images of 194 lipid-related signals of which 93 were uniquely identified or assigned lipid species, see supplemental Table S3. Demographic and histological information of the 12 human carotid plaque specimens can be found in supplemental Table S4 and supplemental Fig. S1; this demonstrates the heterogeneity in histological tissue composition between patients and within patients. Figure 1 illustrates the variety in spatial distributions over six tissue sections, which embody a representative subset of the 106 tissue sections, in terms of histological plaque composition. That is, all representative sections contained NC, four out of six sections contained fibrin areas of variable sizes (section 3, 4, 5, 6), sections 2 and 4 contained a large FC area, and in section 3 a large erythrocyte area was observed. We describe the observations in lipid patterns using these as examples, although the quantitative results we report were obtained on the complete data set. For the masses measured with FTICR we refer to the uploaded data in METASPACE, which is publicly available and can be found as Human CEA Patient I – section 4 (https://tinyurl.com/y4p8prtq), Human CEA Patient H – section 4 (https://tinyurl.com/y2f4jbuv), and Human CEA Patient K – section 8 (https://tinyurl.com/yyqyatod).

**Fig. 2:**
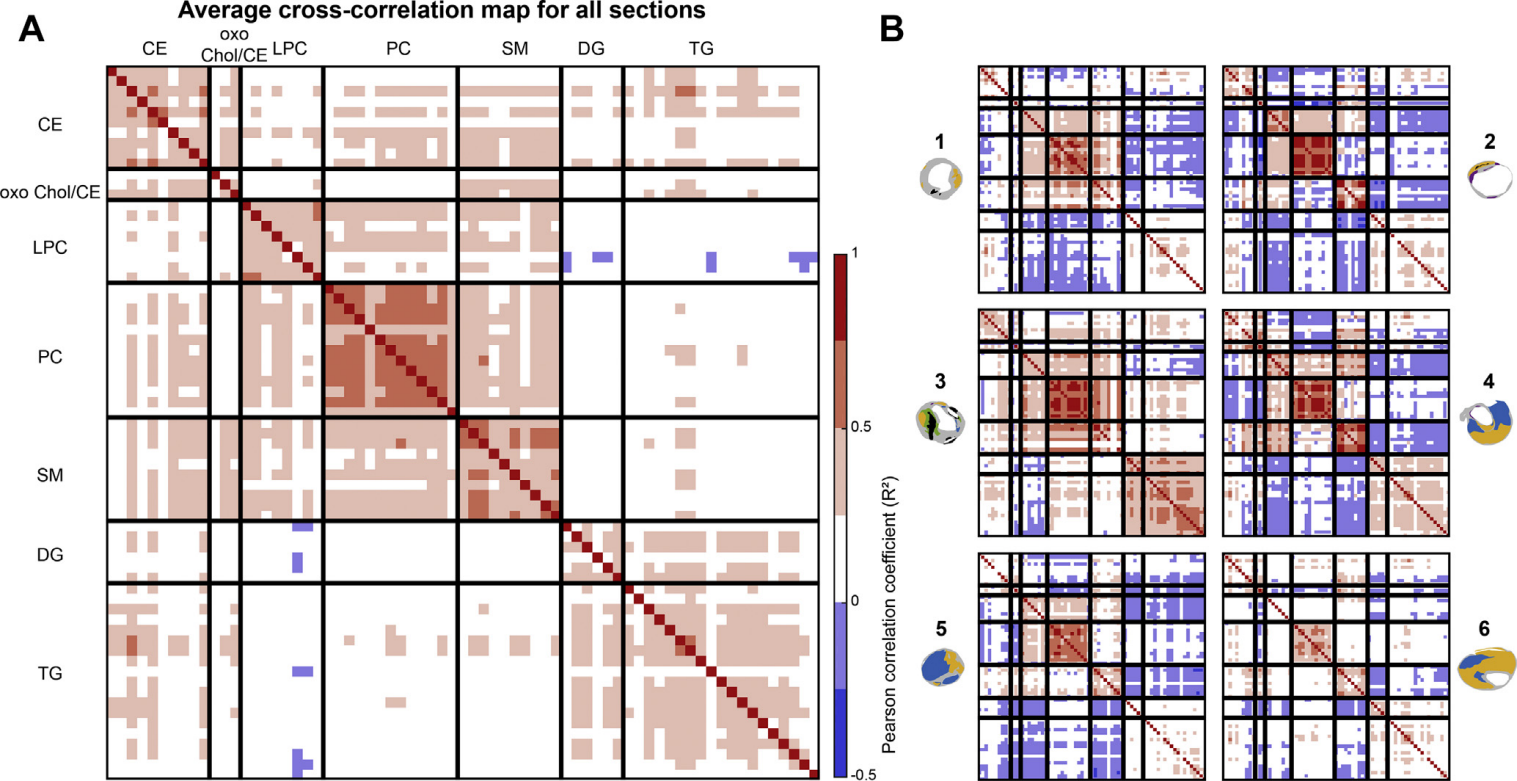
Spatial cross-correlation heatmaps. *x*- and *y*-axes are the same and represent a selection of 70 *m/z* values (supplemental Table S3), classified by lipid class. Per lipid class, the lipids are sorted from low to high *m/z* value. A:. Average heatmap of all 106 tissue sections, showing a correlation within lipid classes and showing a moderate correlation between LPCs, PCs, and SMs and also between DGs and TGs. B:. Individual heatmaps of the six representative sections shown in Fig. 1. Heatmaps of sections 1 and 3 show moderate positive correlation between PC and SM lipids, whereas section 2 shows a negative between these lipids. CE, cholesterol ester; DG, diacylglycerol; LPC, lysophosphatidylcholine; PC, phosphatidylcholines; SM, sphingomyelin; TG, triacylglycerol.

In our dataset we observed lipids belonging to different lipid classes: cholesterol and cholesteryl esters (CEs), lysophosphatidylcholines (LPCs), phosphatidylcholines (PCs), sphingomyelins (SMs), diacylglycerols (DGs), and triacylglycerols (TGs). When stratifying the data by lipid class, we found that lipids belonging to the same lipid classes were distributed similarly over the plaque cross-sections, as quantified by their positive correlation coefficients. The average correlation heatmap of all tissue sections (Fig. 2A) and the heatmaps of the six representative tissue sections (Fig. 2B) are shown.

The cholesteryl ion (*m/z* 369.350) was abundantly present in the mass spectra of all tissue sections. This ion may originate from cholesterol ([M-H_2_O+H]^+^) or as a result of fragmentation of CEs. Its presence was detected over the whole intimal area, although with local variations in intensity. No clear colocalization with histological components was seen. In general, cholesteryl patterns showed moderate cross-correlation with intact CEs (Fig. 2). The spatial patterns of two prominent CEs, CE(18:2) and CE(18:1), show striking differences (Fig. 1 and supplemental Fig. S2). CE(18:2) is often present throughout the cross-section, whereas CE(18:1) is observed in high-intensity spots around the lumen, although not abundant throughout the rest of the tissue sections (Fig. 1). CE lipids in general showed a mild spatial cross-correlation with triacylglycerols (Fig. 2).

**Fig. 3:**
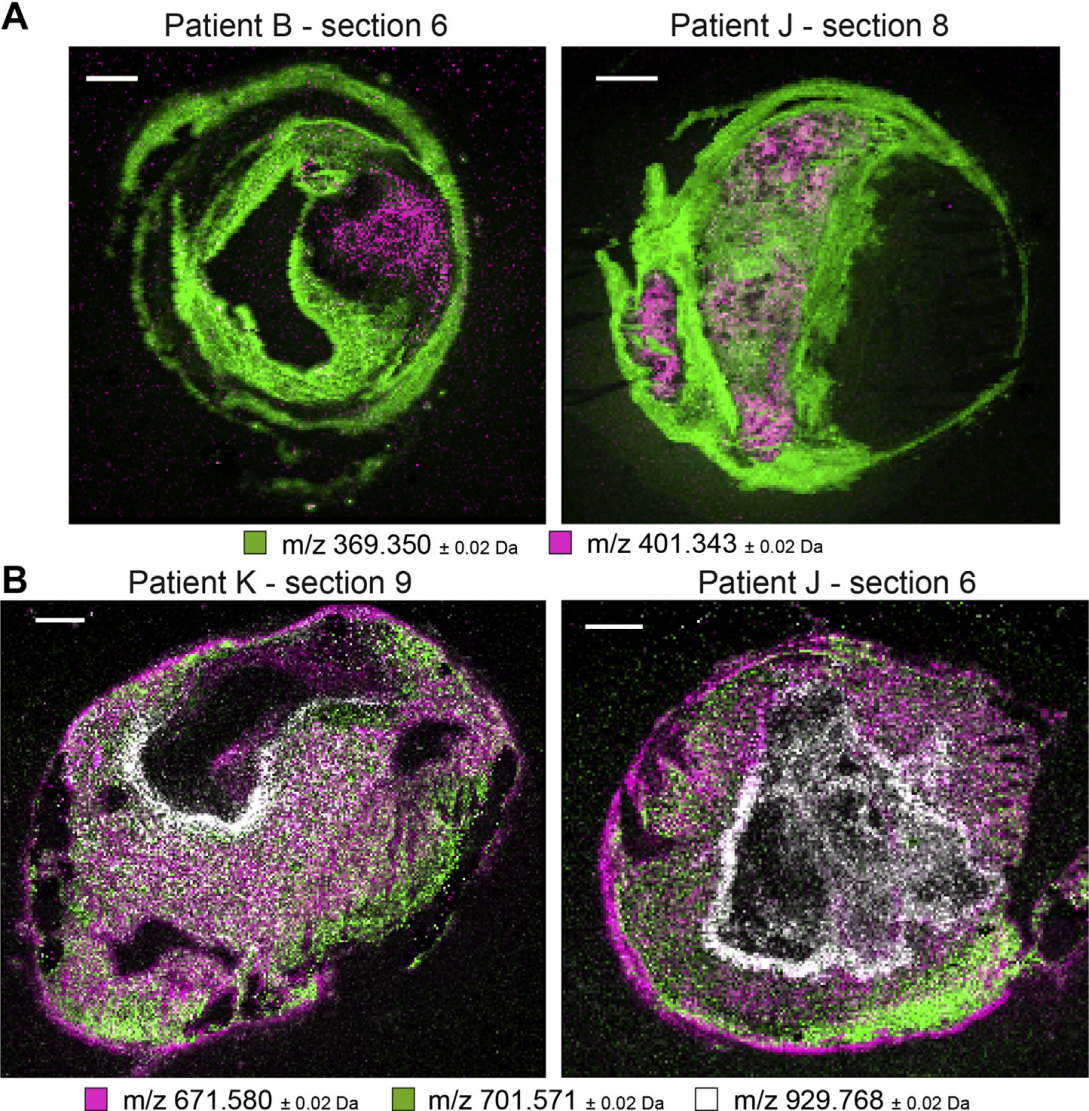
A: Spatial distribution of *m/z* 401.343, annotated as 7-ketocholesterol, in purple, and *m/z* 369.350, identified as cholesteryl ion, in green. 7-Ketocholesterol shows a spatial distribution distinct from that of other lipids in our dataset. B: Combined MALDI-MSI image of spatial distributions of a CE in purple (*m/z* 671.580), SM in green (*m/z* 701.571), and a TG in white (*m/z* 929.767) for three tissue sections. CE is found throughout the tissue section, whereas SM and TG show a more localized spatial pattern. Scale bars are 1 mm.

Although the spatial distribution of most CEs was similar to the distribution of cholesterol itself, one oxidized cholesterol lipid with *m/z* 401.343 was distinct from that of other cholesterol-related molecules (Fig. 2A-oxoChol/CE first row). The spatial location of this species and cholesteryl ion, identified as cholesterol or fragmented cholesterol ester, is depicted in Fig. 3A for two tissue sections. In addition, this molecule showed a unique spatial distribution as it did not show any cross-correlation with other lipids in our dataset and was also not correlated with histological components.

We found that the colocalization of SM, LPC, and PC lipids varied strongly depending on the tissue section. In general, PCs were found to surround the lumen (Fig. 1, sections 2, 6) and sometimes to exude into thrombotic or inflamed areas (Fig. 1, sections 1, 3, 4, 5). In most tissue sections, PC lipids as a group were highly mutually correlated (Fig. 2). The degree of spatial correlation between SMs and PCs was variable between sections, ranging from strong positive correlation, *R*^2^>0.75 (Fig. 2B, sections 1, 3), to negative correlation, *R*^2^<−0.25 (Fig. 2B, sections 2, 4). The distribution of PCs and LPCs was found to be weakly positively correlated in most tissue sections. LPCs are structurally similar to PCs but lack one fatty acid branch. When assessing the tissue sections that showed marked variations in PC and SM distributions (i.e., Fig. 1, sections 2, 4, 5, 6) for the LPC distribution pattern, LPCs were highly abundant at the intersection of the locations that showed high SM and PC intensity.

In contrast, the spatial patterns of most DG and TG lipids showed moderate to high correlation and were distinctive from the distributions of other lipids except for some CEs (Fig. 2B, sections 2, 4, 5, 6). However, the intensities of measured DG were much lower than those of TG. Figure 3B shows overlay images of three lipids identified as a CE, an SM, and a TG for two tissue sections. The variation in spatial distributions between these lipids, which belong to different lipid classes, can clearly be seen.

### The spectral variety in the dataset can be captured by six spectral components

**Fig. 4:**
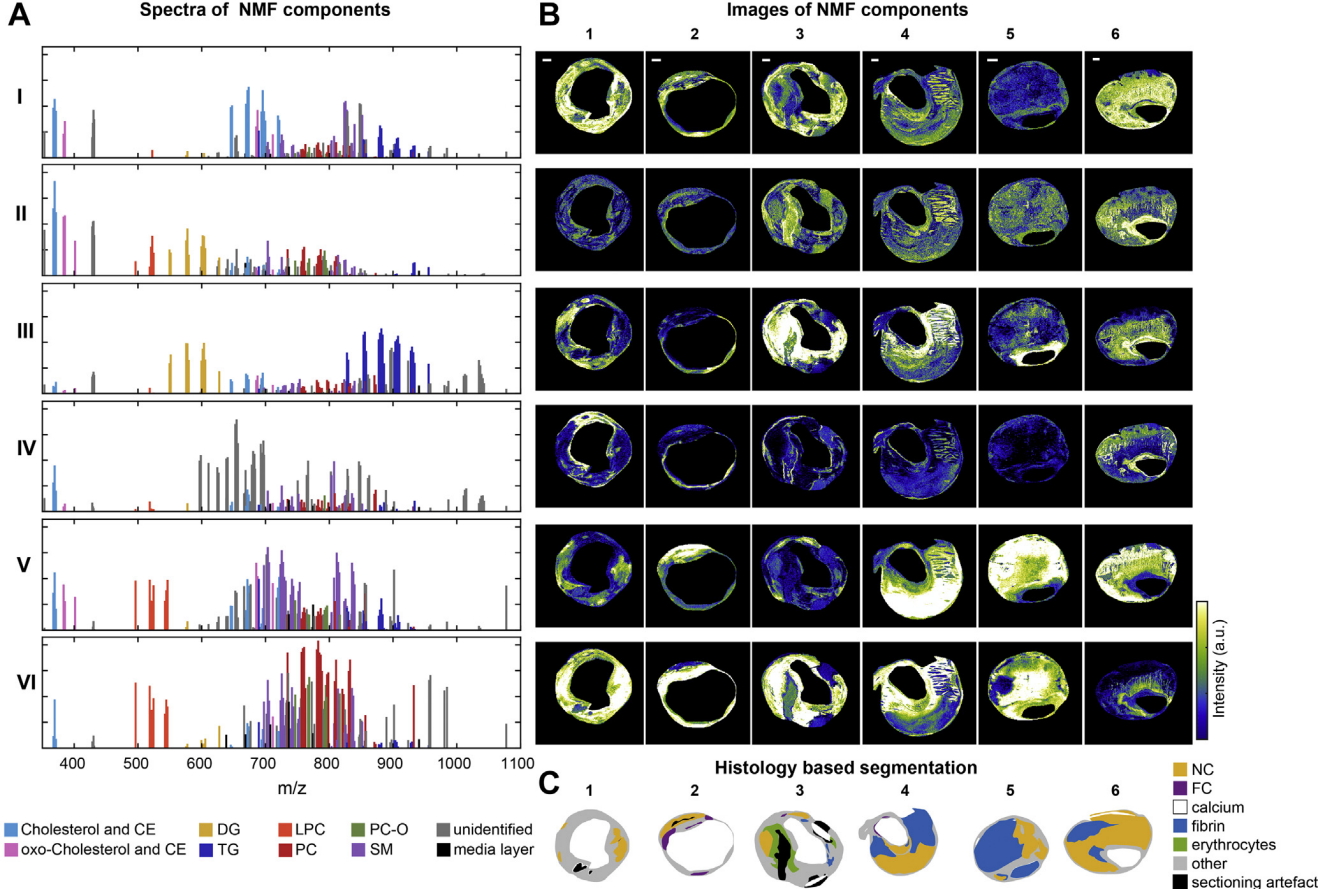
Unsupervised NMF of the 194 *m/z*-containing MALDI-MSI data. A: NMF spectra of the components showing the weight of each *m/z* value to that NMF component. *M/z* values in the NMF component spectra are labeled according to their assigned lipid class, showing a clear separation of lipid classes by means of NMF. B: Corresponding NMF-weighted images of the representative sections, showing the spatial distribution and relative intensities of the NMF components. C: Corresponding histology-based segmentation suggesting colocalization between certain NMF components and histologically relevant features. Scale bars are 1 mm. I–VI, NMF components; 1–6, representative tissue sections (same sections as depicted in Fig. 1); CE, cholesterol ester; DG, diacylglycerol; FC, foam cells; LPC, lysophosphatidylcholines; NC, necrotic core; NMF, nonnegative matrix factorization; PC, phosphatidylcholines; SM, sphingomyelin; TG, triacylglycerol.

We reduced the dimensionality of the spectral data by unsupervised NMF, thereby extracting the major spectral patterns, using six components. Figure 4A and B shows the spectra of the NMF components and the spatial distributions of the six spectral components, respectively. Component I consisted mostly of oxidized and nonoxidized cholesterol and CE lipids. Component II was a combination of cholesterol, both oxidized and nonoxidized, DGs, and a proportion of LPCs. Component III was dominated by DGs and TGs. Component IV represented unidentified *m/z* values. Component V consisted of LPCs, SMs, and cholesterol (esters), both oxidized and nonoxidized, whereas component VI represented the PC moiety of the data.

### Correlation between spatial lipid patterns and histological plaque components

We compared the average spectra of different histological components by fitting a multivariate model. Four multivariate models, discriminating NC, fibrin, FCs, and erythrocytes from all other tissue, were significant, i.e., *R*^2^ > 0.5 and Q^2^ > 0.5, for the combined data of at least six of 12 patients. Model parameters of the significant models are listed in supplemental Table S5. The multivariate models determine the *m/z* values that have the highest influence on separation between two histological components, reported as variable influence for projection (VIP) values. VIP values > 1.0 are considered significant (33), and these are reported in supplemental Table S6. We found SMs and oxidized CEs to be more abundant in NC than in other intima tissue, whereas DGs and TGs were found to be distinctive for areas containing fibrin. The VIP values resulting from the FC model were mostly SMs, whereas the erythrocyte-related VIP values were mostly PCs.

**Fig. 5:**
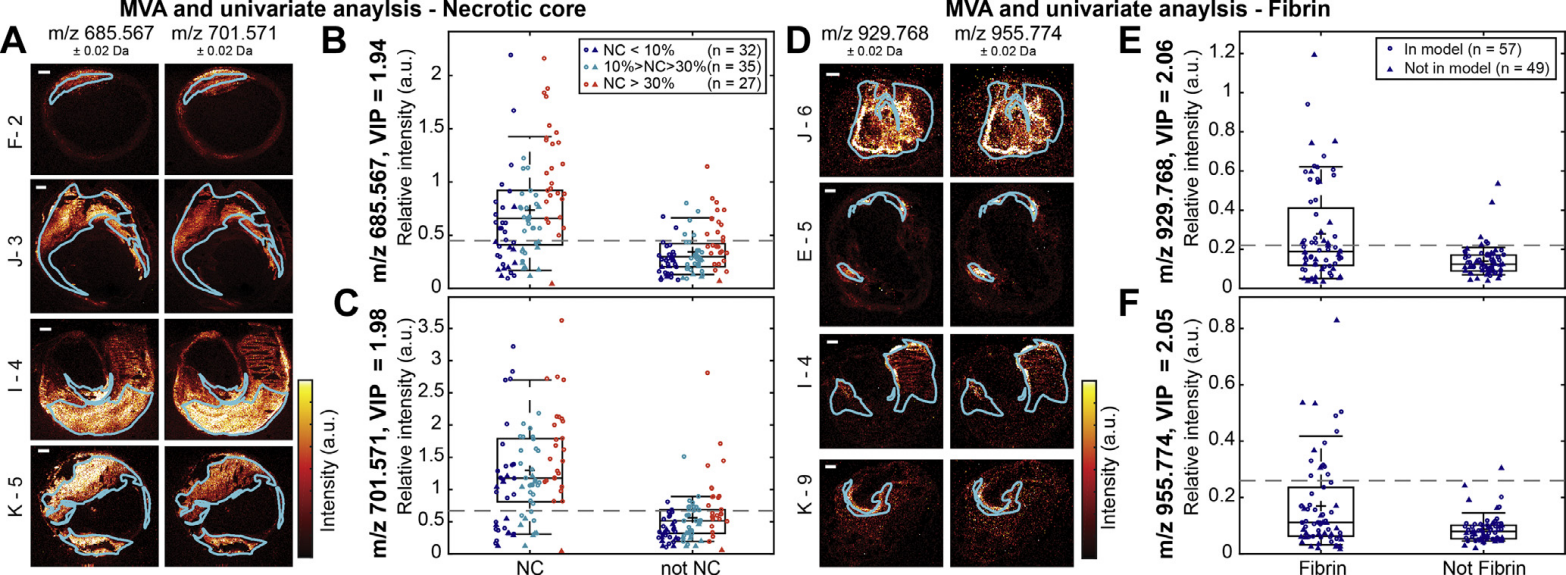
Univariate analysis comparing intensities in necrotic core (NC) versus not-NC areas and fibrin versus not-fibrin areas for two lipids. A: MALDI-MSI images of *m/z* 685.567 (oxoODE-CE [M+Na]^+^) and *m/z* 701.571 (SM(34:2) [M+H]^+^) of four different tissue sections with superimposed outlines of the NC segmentation. Boxplots of *m/z* 685.567 (B) and *m/z* 701.571 (C) showing the mean intensity of the pixels in the NC area and in the not-NC area. Datapoints are colored according to the size of NC present in the corresponding tissue section. D: MALDI-MSI images of *m/z* 929.768 (TG(58:9) [M+H]^+^ or TG(56:6) [M+Na]^+^) and *m/z* 955.774 (TG(60:10) [M+H]^+^ or TG(58:7) [M+Na]^+^) of four different tissue sections with the outlines of the fibrin segmentation. Boxplots of *m/z* 929.768 (E) and *m/z* 955.774 (F) showing the mean intensity of the pixels in the fibrin area and in the not-fibrin area. Only tissue sections containing NC or fibrin were included in these analyses. The dotted lines depict the maximum Youden's index, representing the optimal cutoff intensity value above which the *m/z* value is more likely to be associated with NC or fibrin. For *m/z* 685.567 the threshold is 0.45 and for *m/z* 701.571 it is 0.67, for *m/z* 929.768 it is 0.22 and for *m/z* 955.774 it is 0.26. All *m/z* values showed significantly different intensities between NC and not-NC or between fibrin and not-fibrin (*P*-value < 0.001). ○, data of the patients included in the multivariate model; Δ, data of patients that did not fit the model. Scale bars are 1 mm. Sections F-2 and I-4 are the same sections as 2 and 4 in Figs. 1 and 4, respectively. Sections J-6 and K-9 are also depicted in Fig. 3. MVA, multivariate analysis; NC, necrotic core; VIP, variable influence for projection.

In order to quantitatively establish the contrast in lipid composition between various tissues, we performed two univariate analyses of the intensities of *m/z* values with VIP > 1.0 appearing in the multivariate models: one contrasting NC and not-NC and another contrasting fibrin and not-fibrin. Figure 5 illustrates the results for two *m/z* values. All *m/z* values that had significant VIP values in the multivariate model were significantly (*P*-value < 0.005) different between NC and not-NC in the univariate model, and the difference became stronger with increasing NC sizes. In the univariate analysis of fibrin, 58 of 62 VIPs > 1.0 were significantly different between fibrin and not-fibrin. The four nonsignificant *m/z* values had VIP values lower than 1.14.

### Comparison NMF—multivariate analysis—histology

**Fig. 6:**
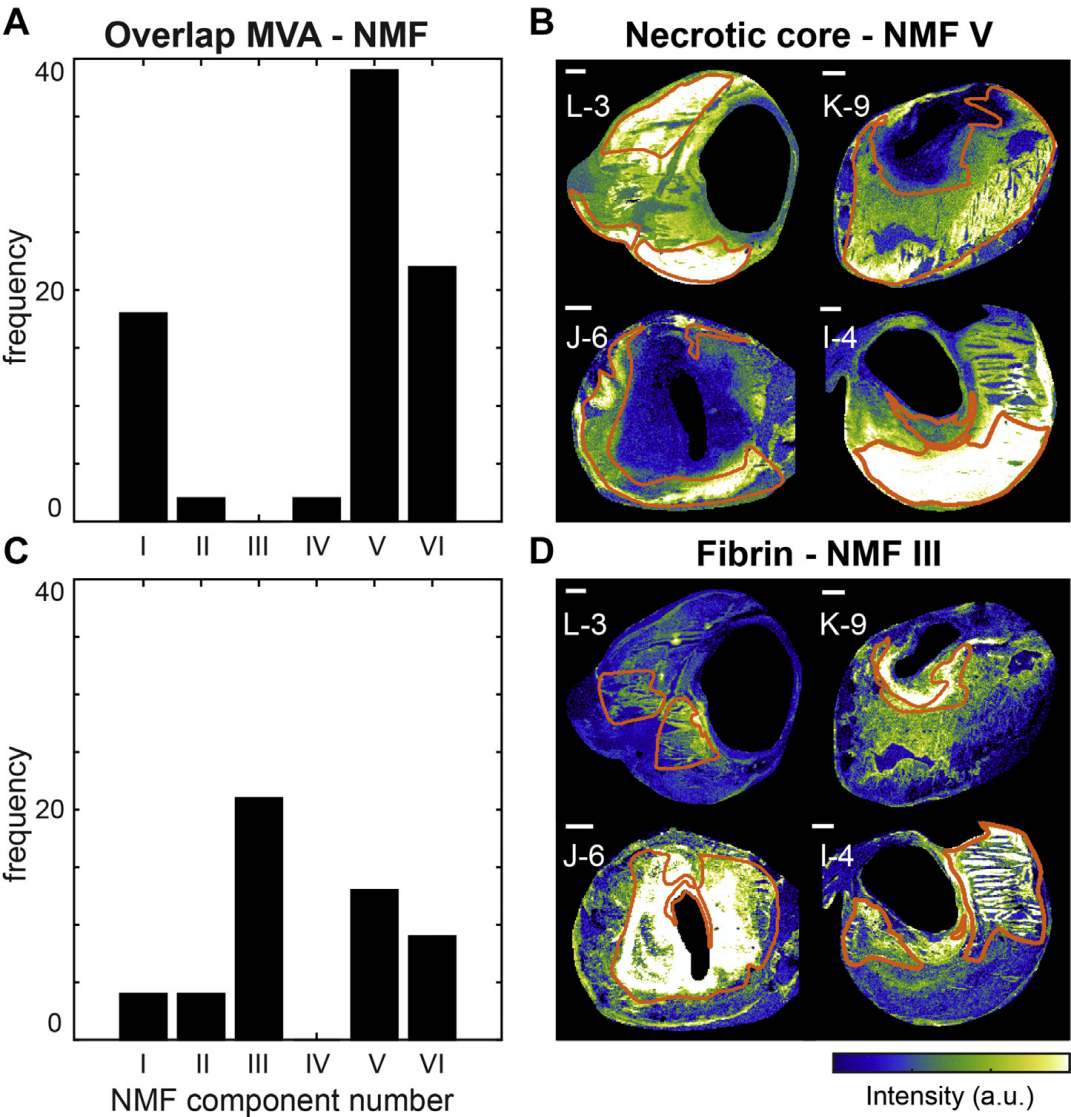
Overview of the correspondence between the histology-based multivariate analysis and the NMF analysis for NC- and fibrin-containing areas. A: Bar graphs showing the number of NC VIP values above 1.0 that were allocated to the different NMF component spectra. Most NC VIP values were allocated to NMF component V. B: The results in A are illustrated by depicting the NMF weight images of component V for four tissue sections with superimposed NC segmentation. C: The number of fibrin VIP values above 1.0 allocated to the different NMF component spectra. Most fibrin VIP values were present in NMF component III. D: The NMF weight images of component III with fibrin segmentation for four tissue sections. Scale bars are 1 mm. Sections J-6 and K-9 are also depicted in Figs. 3 and 5. Section I-4 is also depicted in Figs. 1, 3, 4, and 5. MVA, multivariate analysis; NMF, nonnegative matrix factorization.

Since certain NMF components appeared to colocalize with areas identified in histology (Fig. 4B, C), we investigated the similarities between the NMF spectra and the VIP values that were distinctive for histological components. The majority of the NC *m/z* values with VIP > 1.0 was found in NMF component V, i.e., mostly SMs and oxidized CEs (Fig. 6A).

For fibrin, most VIP *m/z* values were found in NMF component III (Fig. 6C). These VIP values are a set of DG and TG lipids that discriminated areas containing the thrombus-associated protein fibrin.

Figure 6B and D illustrate the correspondence between the histology-based multivariate analysis and the NMF analysis for NC and fibrin areas. The majority of the significant VIP values for erythrocyte areas were most abundant in components VI and III, and the VIP values associated with FCs corresponded to NMF component VI (supplemental Table S7).

## Discussion

With this study, we aimed to visualize the spatial distribution of lipids over the human carotid atherosclerotic plaque and to investigate the relation between lipids and compositional features of plaque vulnerability. We imaged the spatial distribution of 194 lipid-related signals, originating from >90 unique lipid species, in 106 tissue sections of carotid atherosclerotic plaques. This study collects the largest MSI atherosclerosis dataset to date, spatially mapping the molecular lipid content of a series of human plaques, obtaining information on both the cross-sectional and the longitudinal composition of these plaques. Moreover, since we simultaneously processed adjacent tissue sections for histology, this study enabled the comparison of MALDI-MSI results with gold standard histological assessment of plaque stage.

In our dataset we observed lipids belonging to different lipid classes: cholesterol and CEs, LPCs, PCs, SMs, DGs, and TGs. When stratifying the data by lipid class, we found that lipids belonging to the same lipid classes were distributed similarly over the plaque cross-sections, as quantified by their correlation coefficients. PC lipids in particular showed a high mutual spatial correlation. PCs also showed spatial overlap with SMs and LPCs, whereas CEs were spatially correlated to DGs and TGs. However, the degree of spatial correlation was found to differ per tissue section. SMs and oxidized CEs colocalize in NC areas, and this colocalization became more apparent with greater NC size and thus with bigger intima area. The presence of oxidized CEs in NC is in line with theories on pathogenesis of atherosclerosis: during plaque progression, LDL proteins are thought become oxidized and trapped inside the vessel wall (2, 35, 36). The presence of oxidized CE species in plaques, and their increase in abundance with lesion complexity, has been reported previously (12, 37, 38, 39). Increased SM levels in plaques, compared with healthy artery have also been described before (15, 40), as well as the higher abundance of SMs, relative to PCs, with increasing plaque severity (13, 41). Yet, no previous study was able to visualize a clear colocalization of SM and oxidized CE lipid patterns with NC as identified on histology. We showed that the increased SM and oxidized CE levels concentrate in necrotic regions of the plaque, a finding that forms an important addition to the current understanding of the pathophysiology of atherosclerosis, which also provides us with a potential target for detecting NC presence or size in advanced plaques by lipid-sensitive diagnostic imaging technologies (42, 43, 44).

High levels of ceramides in serum, lipids that are structurally related to SMs, have previously been associated with high-risk plaque (45, 46). We detected low levels of ceramides in CEA tissue using the Lipidyzer platform, but ceramide levels were probably below the detection limit of the MALDI-MSI experiments in this study or were suppressed by other ions.

Most cholesterol esters showed a similar spatial distribution. Of interest, however, the spatial distribution of an oxidized cholesterol lipid with *m/z* 401.343 was distinct from that of other cholesterol-related molecules. In addition, this molecule showed a unique spatial distribution as it did not show any cross-correlation with other lipids in our dataset and was also uncorrelated with histological components. According to the literature, *m/z* 401.343 can be annotated as 7-ketocholesterol (14, 47); however, since identification based on MS/MS or chromatographic data is missing, other possible isomers or isobars cannot be excluded. 7-Ketocholesterol has been found in atherosclerotic tissue before (37, 38, 48) and shown to play a proatherogenic role, e.g., by mediating cell death (49, 50).

DGs and TGs showed a spatial distribution that was distinct from that of PCs, SMs, and LPCs. DGs are present in atherosclerotic tissue (51), but they may also form by in-source fragmentation of TGs and PCs. In the spatial NMF analysis, abundant DGs but not TGs and only few PCs appear in NMF component II. Likewise, no DGs are seen in NMF component VI, which features abundant PCs. The observation of these distinct spatial patterns supports the hypothesis that we detect intact species, although partial fragmentation of TGs and PCs cannot be excluded. When comparing the MALDI-MSI images of DG and TG molecules, similarities in the spatial patterns of DG molecules and their presumed TG precursor molecules appear. However, some specific patterns are only visible in the DG images, which confirms the detection of endogenous DG species; see supplemental Fig. S3. Similarly, this occurs for PCs; see supplemental Fig. S4. Our multivariate analysis showed that a set of DG and TG lipids discriminated areas containing the thrombus-associated protein fibrin. A subset of TG lipids was also associated with erythrocyte areas in the multivariate model. Previously (19), we described a colocalization between DGs and thrombus and this observation is thus confirmed in our current dataset. We hypothesize that the colocalization of DGs and thrombotic plaque elements can be explained by the role that DGs play in regulating protein kinase C, an enzyme known to induce an array of proatherogenic responses (52), including impairment of fibrinolysis (53) and platelet activation (54, 55).

The tissue areas that contained FCs and erythrocytes were generally smaller than those of NC and fibrin. The lipids that were found to be distinctive for FC areas in the multivariate model were mostly PCs. When comparing the FC VIP values and the NMF component spectra, the majority of FC VIPs were present in the PC-dominated NMF component VI. Thus, there was a large spatial overlap between the outcomes of the histology-based multivariate model and the NMF classification (Fig. 6). A subset of erythrocyte-associated *m/z* values—as found by the multivariate analysis—were also PC lipids. PC lipids are major constituents of healthy cell membranes (56), which probably explains why the spatial distribution of PC lipids was found to correlate with areas of high cellularity, for instance, FC- and erythrocyte-rich areas.

### Limitations

In order to facilitate processing of a large number of sections, we opted to perform experiments in positive mode only. Lipids that have been associated with atherosclerosis previously, e.g., SMs (13, 14, 15, 57, 58, 59), CEs (9, 12, 15, 18, 58, 60, 61, 62), and PCs (9, 12, 13, 14, 15, 18, 58, 60, 61, 63), have been shown to be detectable in positive mode.

Lipid identities could not be assigned to a subset of the 194 *m/z* values that were measured in the MALDI-MSI experiments. Most of the unknown masses in the MALDI-MSI data were not detected in the data captured by the Lipidyzer and FTICR measurements that were used for mass identification purposes. In addition, these unknown *m/z* values were not reported in the databases we consulted (Human Metabolome Database, SwissLipids, and LipidMaps). The unidentified *m/z* values were dominating the spectrum of NMF component IV, while barely contributing to the spectra of the other NMF components. The distribution images of NMF component IV indicate that the unidentified *m/z* values were mostly present at the edges of the MALDI-MSI cross-sections. We assume that these images represent artifacts of the MALDI-MSI measurement method.

In the annotation by exact mass in FTICR, a few isobaric species are candidates for assignment. In addition to mass, we took into account the relative sensitivity of MALDI-MSI and detection likelihood in confidently annotating these peaks.

For the Synapt data, we found a mass error of on average 7.02 ppm with a standard deviation of 4.40 when calculating the mass error based on the exact mass of the identified lipids. To strengthen lipid identification, we have tried to perform MS/MS analysis on tissue for lipid identification; however, our system was lacking sensitivity for the lipids that are ambiguous. We therefore chose to use the Lipidyzer platform and the FTICR system to get the best possible annotation and identification. The mass error of lipids annotated from the FTICR measurement is on average 0.11 ppm with a standard deviation of 0.015.

## Conclusion

In our endeavor to identify lipids characteristic of high-risk plaque phenotype, we conclude that certain SMs and oxidized CEs are significantly higher expressed in NC and that the abundance of these molecules is correlated to the size of the NC. Interesting observations to build further studies on are the correlation between DGs and TGs and thrombus fragments, i.e., erythrocytes and fibrin, providing a possible marker for intraplaque bleeding. In addition, erythrocytes and FCs are correlated with PCs. These findings add to the current understanding of atherosclerosis pathogenesis. In the future, these marker lipids related to “vulnerable” plaque features may serve as targets for in vivo imaging and therapeutic applications, aiding the lipidomic identification of plaques, and thus patients, in need of focal or systemic preventive treatment.

### Data availability

All data are provided within the main text and supplemental document. FTICR data are available in METASPACE, as Human CEA Patient I – section 4 and Human CEA Patient H – section 4. Raw data are available on request.

## Conflict of interest

The authors declare that they have no conflicts of interest with the contents of this article.
