# Supplementary material for: Lipid signature of advanced human carotid atherosclerosis assessed by mass spectrometry imaging
Source: J Lipid Res. 2021 Jan 6;62:100020. doi: 10.1194/jlr.RA120000974 (PMC7881220; doi:10.1194/jlr.RA120000974)
Supplement: Supplemental Material [file mmc1.docx]

**Supplemental Material**

**Lipid signature of advanced human carotid atherosclerosis assessed by mass spectrometry imaging**

Astrid M. Moerman^1,#^ , Mirjam Visscher^1,#^ , Nuria Slijkhuis^1^, Kim Van Gaalen^1^, Bram Heijs^2^, Theo Klein^3^, Peter C. Burgers^4^, Yolanda B. De Rijke^3^, Heleen M.M. Van Beusekom^5^, Theo M. Luider^4^, Hence J.M. Verhagen^6^, Antonius F.W. Van der Steen^1,7^ , Frank J.H. Gijsen^1^, Kim Van der Heiden^1,&^ , Gijs Van Soest^1,&,*^

1 Department of Cardiology, Erasmus MC University Medical Center Rotterdam, Rotterdam, The Netherlands

2 Center for Proteomics and Metabolomics, Leiden University Medical Center, Leiden, The Netherlands

3 Department of Clinical Chemistry, Erasmus MC University Medical Center Rotterdam, Rotterdam, The Netherlands

4 Department of Neurology, Laboratory of Neuro-Oncology, Erasmus MC University Medical Center Rotterdam, Rotterdam, The Netherlands

5 Department of Experimental Cardiology, Erasmus MC University Medical Center Rotterdam, Rotterdam, The Netherlands

6 Department of Vascular and Endovascular Surgery, Erasmus MC University Medical Center Rotterdam, Rotterdam, The Netherlands

7 Shenzhen Institutes of Advanced Technology, Chinese Academy of Sciences, Shenzhen, China

# these authors contributed equally

& these authors contributed equally

*Corresponding author: Gijs van Soest
E-mail: g.vansoest@erasmusmc.nl
Postal address: P.O. Box 2040, 3000 CA Rotterdam, The Netherlands
Telephone number: +31 (0) 10 704 46 38

| **Content** |  | **Page** |
| --- | --- | --- |
| Supplemental Table S1 | Lipidyzer SRM scan transitions | 2 |
| Supplemental Table S2 | Lipid class concentration and composition of the Lipidyzer samples | 5 |
| Supplemental Table S3 | List of 194 lipids measured in human carotid atherosclerotic plaques | 6 |
| Supplemental Table S4 | Demographic information a histological tissue compositions per patient | 9 |
| Supplemental Figure S1 | Histological tissue composition of 12 carotid plaques | 10 |
| Supplemental Figure S2 | Combined MALDI-MSI image of CE(18:1) and CE(18:2) | 11 |
| Supplemental Figure S3 | Comparison of spatial patterns of DG and TG molecules | 12 |
| Supplemental Figure S4 | Comparison of spatial patterns of DG and PC molecules | 13 |
| Supplemental Table S5 | OPLS-DA model parameters | 14 |
| Supplemental Table S6 | Lists of *m/z* values with VIP > 1.0 resulting from significant OPLS-DA models | 15 |
| Supplemental Table S7 | Number of *m/z* values with VIP > 1.0 for the different multivariate models | 19 |
| Supplemental References | | 20 |

**Supplemental Table S1.** Lipidyzer SRM scan transitions

| **Lipid ID** | **Q1 Mass (Da)** | **Q2 Mass (Da)** |  | **Lipid ID** | **Q1 Mass (Da)** | **Q2 Mass (Da)** |
| --- | --- | --- | --- | --- | --- | --- |
| PC(12:0/14:1) | 706.500 | 225.200 |  | PC(14:0/22:2) | 844.600 | 335.300 |
| PC(16:0/12:0) | 736.500 | 255.200 |  | PC(14:0/22:4) | 840.600 | 331.300 |
| PC(12:0/16:1) | 734.500 | 253.200 |  | PC(14:0/22:5) | 838.600 | 329.200 |
| PC(18:0/12:0) | 764.500 | 283.300 |  | PC(14:0/22:6) | 836.500 | 327.200 |
| PC(12:0/18:1) | 762.500 | 281.200 |  | PC(15:0/18:2) | 802.600 | 241.200 |
| PC(12:0/18:2) | 760.500 | 279.200 |  | PC(16:0/18:0) | 820.600 | 283.300 |
| PC(12:0/18:3) | 758.500 | 277.200 |  | PC(16:0/20:3) | 842.600 | 305.200 |
| PC(12:0/18:4) | 756.500 | 275.200 |  | PC(16:0/22:5) | 866.600 | 329.200 |
| PC(12:0/20:1) | 790.600 | 309.300 |  | PC(16:0/22:6) | 864.600 | 327.200 |
| PC(12:0/20:2) | 788.500 | 307.300 |  | PC(17:0/18:1) | 832.600 | 269.200 |
| PC(12:0/20:3) | 786.500 | 305.200 |  | PC(17:0/18:2) | 830.600 | 269.200 |
| PC(12:0/20:4) | 784.500 | 303.200 |  | PC(18:0/18:0) | 848.600 | 283.300 |
| PC(12:0/20:5) | 782.500 | 301.200 |  | PC(18:0/22:6) | 892.600 | 327.200 |
| PC(12:0/22:2) | 816.600 | 335.300 |  | PC(18:1/22:6) | 890.600 | 327.200 |
| PC(12:0/22:4) | 812.500 | 331.300 |  | PC(18:2/20:5) | 862.600 | 301.200 |
| PC(12:0/22:5) | 810.500 | 329.200 |  | PC(18:2/22:6) | 888.600 | 327.200 |
| PC(12:0/22:6) | 808.500 | 327.200 |  | dPC(16:0/16:1) | 799.700 | 253.200 |
| PC(15:0/14:1) | 748.500 | 241.200 |  | dPC(16:0/18:1) | 827.600 | 281.200 |
| PC(15:0/16:1) | 776.500 | 241.200 |  | dPC(16:0/18:2) | 825.600 | 279.200 |
| PC(15:0/18:3) | 800.500 | 241.200 |  | dPC(16:0/18:3) | 823.600 | 277.200 |
| PC(15:0/20:3) | 828.600 | 241.200 |  | dPC(16:0/20:3) | 851.600 | 305.200 |
| PC(15:0/20:4) | 826.600 | 241.200 |  | dPC(16:0/20:4) | 849.600 | 303.200 |
| PC(15:0/20:5) | 824.500 | 241.200 |  | dPC(16:0/20:5) | 847.600 | 301.200 |
| PC(15:0/22:4) | 854.600 | 241.200 |  | dPC(16:0/22:4) | 877.600 | 331.200 |
| PC(15:0/22:5) | 852.600 | 241.200 |  | dPC(16:0/22:5) | 875.600 | 329.200 |
| PC(15:0/22:6) | 850.600 | 241.200 |  | dPC(16:0/22:6) | 873.600 | 327.200 |
| PC(16:0/22:1) | 874.700 | 337.300 |  | PE(14:0/14:0) | 634.400 | 227.200 |
| PC(17:0/16:1) | 804.600 | 269.200 |  | PE(14:0/14:1) | 632.400 | 225.200 |
| PC(17:0/20:3) | 856.600 | 269.200 |  | PE(16:0/14:0) | 662.500 | 255.200 |
| PC(17:0/22:4) | 882.600 | 269.200 |  | PE(14:0/16:1) | 660.500 | 253.200 |
| PC(17:0/22:5) | 880.600 | 269.200 |  | PE(18:0/14:0) | 690.500 | 283.300 |
| PC(17:0/22:6) | 878.600 | 269.200 |  | PE(14:0/18:1) | 688.500 | 281.200 |
| PC(18:0/16:1) | 818.600 | 253.200 |  | PE(14:0/18:2) | 686.500 | 279.200 |
| PC(20:0/16:1) | 846.600 | 253.200 |  | PE(14:0/18:3) | 684.500 | 277.200 |
| PC(18:0/20:0) | 876.700 | 283.300 |  | PE(14:0/18:4) | 682.400 | 275.200 |
| PC(20:0/18:1) | 874.600 | 281.200 |  | PE(14:0/20:1) | 716.500 | 309.300 |
| PC(20:0/18:2) | 872.600 | 279.200 |  | PE(14:0/20:2) | 714.500 | 307.300 |
| PC(20:0/18:3) | 870.600 | 277.200 |  | PE(14:0/20:3) | 712.500 | 305.200 |
| PC(20:0/18:4) | 868.600 | 275.200 |  | PE(14:0/20:4) | 710.500 | 303.200 |
| PC(20:0/20:1) | 902.700 | 309.300 |  | PE(14:0/20:5) | 708.500 | 301.200 |
| PC(20:0/20:2) | 900.700 | 307.300 |  | PE(14:0/22:1) | 744.600 | 337.300 |
| PC(20:0/20:3) | 898.700 | 305.200 |  | PE(14:0/22:2) | 742.500 | 335.300 |
| PC(20:0/20:4) | 896.600 | 303.200 |  | PE(14:0/22:4) | 738.500 | 331.300 |
| PC(20:0/20:5) | 894.600 | 301.200 |  | PE(15:0/14:1) | 646.400 | 241.200 |
| PC(20:0/22:2) | 928.700 | 335.300 |  | PE(16:0/15:0) | 676.500 | 241.200 |
| PC(20:0/22:4) | 924.700 | 331.300 |  | PE(15:0/16:1) | 674.500 | 241.200 |
| PC(20:0/22:5) | 922.700 | 329.200 |  | PE(18:0/15:0) | 704.500 | 241.200 |
| PC(20:0/22:6) | 920.600 | 327.200 |  | PE(15:0/18:1) | 702.500 | 241.200 |
| PC(18:0/14:0) | 792.600 | 227.200 |  | PE(15:0/18:2) | 700.500 | 241.200 |
| PC(14:0/20:3) | 814.600 | 305.200 |  | PE(15:0/18:3) | 698.500 | 241.200 |
| **Lipid ID** | **Q1 Mass (Da)** | **Q2 Mass (Da)** |  | **Lipid ID** | **Q1 Mass (Da)** | **Q2 Mass (Da)** |
| PE(15:0/18:4) | 696.500 | 241.200 |  | dPE(18:0/20:5) | 769.500 | 301.200 |
| PE(15:0/20:2) | 728.500 | 241.200 |  | dPE(18:0/22:5) | 797.600 | 329.200 |
| PE(15:0/20:3) | 726.500 | 241.200 |  | dPE(18:0/22:6) | 795.500 | 327.200 |
| PE(15:0/20:4) | 724.500 | 241.200 |  | LPC(12:0) | 498.300 | 199.200 |
| PE(15:0/20:5) | 722.500 | 241.200 |  | LPC(14:0) | 526.300 | 227.200 |
| PE(15:0/22:1) | 758.600 | 241.200 |  | LPC(14:1) | 524.300 | 225.200 |
| PE(15:0/22:2) | 756.600 | 241.200 |  | LPC(15:0) | 540.300 | 241.200 |
| PE(15:0/22:4) | 752.500 | 241.200 |  | LPC(16:0) | 554.300 | 255.200 |
| PE(15:0/22:5) | 750.500 | 241.200 |  | LPC(16:1) | 552.300 | 253.200 |
| PE(18:0/16:0) | 718.500 | 283.300 |  | dLPC(16:0) | 563.200 | 264.200 |
| PE(16:0/20:3) | 740.500 | 305.200 |  | LPC(17:0) | 568.300 | 269.200 |
| PE(16:0/22:1) | 772.600 | 337.300 |  | LPC(18:0) | 582.400 | 283.300 |
| PE(16:0/22:2) | 770.600 | 335.300 |  | LPC(18:1) | 580.400 | 281.200 |
| PE(16:0/22:4) | 766.500 | 331.300 |  | LPC(18:2) | 578.300 | 279.200 |
| PE(18:0/17:0) | 732.600 | 269.200 |  | LPC(18:3) | 576.300 | 277.200 |
| PE(17:0/18:1) | 730.500 | 269.200 |  | LPC(18:4) | 574.300 | 275.200 |
| PE(17:0/20:3) | 754.500 | 269.200 |  | LPC(20:0) | 610.400 | 311.300 |
| PE(17:0/22:1) | 786.600 | 269.200 |  | LPC(20:1) | 608.400 | 309.300 |
| PE(17:0/22:2) | 784.600 | 269.200 |  | LPC(20:2) | 606.400 | 307.300 |
| PE(17:0/22:4) | 780.600 | 269.200 |  | LPC(20:3) | 604.400 | 305.200 |
| PE(17:0/22:5) | 778.500 | 269.200 |  | LPC(20:4) | 602.400 | 303.200 |
| PE(18:0/18:0) | 746.600 | 283.300 |  | LPC(20:5) | 600.300 | 301.200 |
| PE(18:0/20:3) | 768.600 | 305.200 |  | LPC(22:0) | 638.400 | 339.300 |
| PE(18:0/22:2) | 798.600 | 335.300 |  | LPC(22:1) | 636.400 | 337.300 |
| PE(18:0/22:4) | 794.600 | 331.300 |  | LPC(22:4) | 630.400 | 331.300 |
| PE(18:0/22:6) | 790.500 | 327.200 |  | LPC(22:5) | 628.400 | 329.200 |
| PE(22:0/18:1) | 800.600 | 281.200 |  | LPC(22:6) | 626.300 | 327.200 |
| PE(18:1/22:2) | 796.600 | 335.300 |  | LPC(24:0) | 666.500 | 367.400 |
| PE(O-16:0/14:1) | 646.500 | 225.200 |  | LPC(24:1) | 664.500 | 365.300 |
| PE(O-16:0/18:0) | 704.600 | 283.300 |  | LPE(12:0) | 396.200 | 199.200 |
| PE(O-16:0/20:1) | 730.600 | 309.300 |  | LPE(14:0) | 424.200 | 227.200 |
| PE(O-16:0/20:2) | 728.600 | 307.300 |  | LPE(14:1) | 422.200 | 225.200 |
| PE(O-16:0/22:4) | 752.600 | 331.300 |  | LPE(15:0) | 438.300 | 241.200 |
| PE(O-18:0/20:3) | 754.600 | 305.200 |  | LPE(16:0) | 452.300 | 255.200 |
| PE(O-18:0/22:5) | 778.600 | 329.200 |  | LPE(16:1) | 450.300 | 253.200 |
| PE(O-18:0/22:6) | 776.600 | 327.200 |  | LPE(17:0) | 466.300 | 269.200 |
| PE(P-14:0/18:1) | 672.500 | 281.200 |  | LPE(18:0) | 480.300 | 283.300 |
| PE(P-14:1/18:1) | 670.500 | 281.200 |  | LPE(18:1) | 478.300 | 281.200 |
| PE(P-16:0/14:1) | 644.500 | 225.200 |  | LPE(18:2) | 476.300 | 279.200 |
| PE(P-16:0/18:4) | 694.500 | 275.200 |  | LPE(18:3) | 474.300 | 277.200 |
| PE(P-16:0/20:5) | 720.500 | 301.200 |  | LPE(18:4) | 472.200 | 275.200 |
| PE(P-16:0/22:6) | 746.500 | 327.200 |  | LPE(20:0) | 508.300 | 311.300 |
| PE(P-18:0/22:2) | 782.600 | 335.300 |  | LPE(20:1) | 506.300 | 309.300 |
| PE(P-18:0/22:6) | 774.500 | 327.200 |  | LPE(20:2) | 504.300 | 307.300 |
| PE(P-18:1/22:6) | 772.500 | 327.200 |  | LPE(20:3) | 502.300 | 305.200 |
| PE(P-18:2/22:6) | 770.500 | 327.200 |  | LPE(20:4) | 500.300 | 303.200 |
| dPE(18:0/18:1) | 749.600 | 281.200 |  | LPE(22:0) | 536.400 | 339.300 |
| dPE(18:0/18:2) | 747.500 | 279.200 |  | LPE(22:1) | 534.400 | 337.300 |
| dPE(18:0/18:3) | 745.500 | 277.200 |  | LPE(22:2) | 532.300 | 335.300 |
| dPE(18:0/20:3) | 773.600 | 305.200 |  | LPE(22:4) | 528.300 | 331.300 |
| dPE(18:0/20:4) | 771.500 | 303.200 |  | LPE(24:0) | 564.400 | 367.400 |
| **Lipid ID** | **Q1 Mass (Da)** | **Q2 Mass (Da)** |  |  |  |  |
| LPE(24:1) | 562.400 | 365.300 |  |  |  |  |
| dLPE(18:0) | 485.300 | 288.300 |  |  |  |  |
| SM(14:0) | 675.500 | 184.100 |  |  |  |  |
| SM(16:0) | 703.600 | 184.100 |  |  |  |  |
| SM(18:0) | 731.600 | 184.100 |  |  |  |  |
| SM(18:1) | 729.600 | 184.100 |  |  |  |  |
| SM(20:0) | 759.600 | 184.100 |  |  |  |  |
| SM(20:1) | 757.600 | 184.100 |  |  |  |  |
| SM(22:0) | 787.700 | 184.100 |  |  |  |  |
| SM(22:1) | 785.700 | 184.100 |  |  |  |  |
| SM(24:0) | 815.700 | 184.100 |  |  |  |  |
| SM(24:1) | 813.700 | 184.100 |  |  |  |  |
| SM(26:0) | 843.700 | 184.100 |  |  |  |  |
| SM(26:1) | 841.700 | 184.100 |  |  |  |  |
| dSM(16:0) | 710.600 | 184.200 |  |  |  |  |
| dSM(18:1) | 736.600 | 184.200 |  |  |  |  |
| dSM(24:0) | 822.700 | 184.200 |  |  |  |  |
| dSM(24:1) | 820.700 | 184.200 |  |  |  |  |
| FFA(12:0) | 199.200 | 199.200 |  |  |  |  |
| FFA(14:0) | 227.200 | 227.200 |  |  |  |  |
| FFA(14:1) | 225.200 | 225.200 |  |  |  |  |
| FFA(15:0) | 241.200 | 241.200 |  |  |  |  |
| FFA(16:0) | 255.200 | 255.200 |  |  |  |  |
| FFA(16:1) | 253.200 | 253.200 |  |  |  |  |
| FFA(17:0) | 269.200 | 269.200 |  |  |  |  |
| FFA(18:0) | 283.300 | 283.300 |  |  |  |  |
| FFA(18:1) | 281.200 | 281.200 |  |  |  |  |
| FFA(18:2) | 279.200 | 279.200 |  |  |  |  |
| FFA(18:3) | 277.200 | 277.200 |  |  |  |  |
| FFA(18:4) | 275.200 | 275.200 |  |  |  |  |
| FFA(20:0) | 311.300 | 311.300 |  |  |  |  |
| FFA(20:1) | 309.300 | 309.300 |  |  |  |  |
| FFA(20:2) | 307.300 | 307.300 |  |  |  |  |
| FFA(20:3) | 305.200 | 305.200 |  |  |  |  |
| FFA(20:4) | 303.200 | 303.200 |  |  |  |  |
| FFA(20:5) | 301.200 | 301.200 |  |  |  |  |
| FFA(22:0) | 339.300 | 339.300 |  |  |  |  |
| FFA(22:1) | 337.300 | 337.300 |  |  |  |  |
| FFA(22:2) | 335.300 | 335.300 |  |  |  |  |
| FFA(22:4) | 331.300 | 331.300 |  |  |  |  |
| FFA(22:5) | 329.200 | 329.200 |  |  |  |  |
| FFA(22:6) | 327.200 | 327.200 |  |  |  |  |
| FFA(24:0) | 367.400 | 367.400 |  |  |  |  |
| FFA(24:1) | 365.300 | 365.300 |  |  |  |  |
| dFFA(16:0) | 264.200 | 264.200 |  |  |  |  |
| dFFA(17:1) | 267.200 | 267.200 |  |  |  |  |

**Supplemental Table S2.** Concentration (µg/L) and composition (%) of the lipid classes per piece (n=9) of carotid artery originating from the same CEA sample used for the Lipidyzer analysis. CE: cholesteryl ester, CER: Ceramide, DG: diacylglycerol, DCER: dihydroceramides, FFA: Free Fatty Acids, HCER: hexosylceramides, LCER: lactosylceramides, PC: phosphatidylcholines, SM: sphingomyelin, TG: triacylglycerol

| **Sample** | **CE** | | **CER** | | **DG** | | **DCER** | | **FFA** | |
| --- | --- | --- | --- | --- | --- | --- | --- | --- | --- | --- |
| **#** | **µg/L** | **%** | **µg/L** | **%** | **µg/L** | **%** | **µg/L** | **%** | **µg/L** | **%** |
| 1 | 21.18 | 3.96 | 4.75 | 0.89 | 5.08 | 0.95 | 1.02 | 0.19 | 179.30 | 33.49 |
| 2 | 32.47 | 6.19 | 5.02 | 0.96 | 11.55 | 2.20 | 1.09 | 0.21 | 110.50 | 21.05 |
| 3 | 54.25 | 6.06 | 8.80 | 0.98 | 23.71 | 2.65 | 1.30 | 0.15 | 172.00 | 19.20 |
| 4 | 77.15 | 5.99 | 13.35 | 1.04 | 32.39 | 2.51 | 1.87 | 0.15 | 210.85 | 16.37 |
| 5 | 27.03 | 5.25 | 4.10 | 0.80 | 9.66 | 1.88 | 0.74 | 0.14 | 108.52 | 21.08 |
| 6 | 31.58 | 5.52 | 4.30 | 0.75 | 12.06 | 2.11 | 1.01 | 0.18 | 119.57 | 20.88 |
| 7 | 35.46 | 6.43 | 4.53 | 0.82 | 21.24 | 3.85 | 0.94 | 0.17 | 121.55 | 22.03 |
| 8 | 23.01 | 4.55 | 2.91 | 0.58 | 9.02 | 1.78 | 0.63 | 0.13 | 100.44 | 19.87 |
| 9 | 29.08 | 5.09 | 3.68 | 0.64 | 11.57 | 2.02 | 0.95 | 0.17 | 118.37 | 20.72 |
| **Sample** | **HCER** | | **LCER** | | **PC** | | **SM** | | **TG** | |
| **#** | **µg/L** | **%** | **µg/L** | **%** | **µg/L** | **%** | **µg/L** | **%** | **µg/L** | **%** |
| 1 | 5.07 | 0.95 | 1.83 | 0.34 | 56.08 | 10.48 | 226.16 | 42.25 | 34.86 | 6.51 |
| 2 | 4.49 | 0.85 | 2.18 | 0.42 | 54.90 | 10.46 | 242.68 | 46.22 | 60.13 | 11.45 |
| 3 | 8.85 | 0.99 | 5.29 | 0.59 | 116.96 | 13.06 | 370.15 | 41.32 | 134.60 | 15.02 |
| 4 | 11.30 | 0.88 | 6.39 | 0.50 | 141.31 | 10.97 | 463.22 | 35.96 | 330.47 | 25.65 |
| 5 | 3.22 | 0.62 | 2.62 | 0.51 | 60.10 | 11.67 | 201.84 | 39.21 | 96.99 | 18.84 |
| 6 | 3.58 | 0.63 | 3.00 | 0.52 | 71.28 | 12.45 | 213.27 | 37.25 | 112.91 | 19.72 |
| 7 | 4.43 | 0.80 | 3.33 | 0.60 | 43.00 | 7.79 | 223.23 | 40.45 | 94.14 | 17.06 |
| 8 | 2.66 | 0.53 | 2.60 | 0.51 | 64.97 | 12.85 | 194.36 | 38.45 | 104.91 | 20.75 |
| 9 | 3.92 | 0.69 | 2.61 | 0.46 | 83.02 | 14.53 | 243.59 | 42.63 | 74.57 | 13.05 |

**Supplemental Table S3.** List of 194 lipids measured in human carotid atherosclerotic plaques

| ***m/z ± 0.02*** | **lipid class** | **ID** | **adduct** | ***m/z ± 0.02*** | **lipid class** | **ID** | **adduct** |
| --- | --- | --- | --- | --- | --- | --- | --- |
| 353.331 | unknown |  |  | 638.472 | unknown |  |  |
| 367.343 | Chol | Cholesterol derivative^LM,d^ | [M-H2O+H]+ | 638.587 | unknown |  |  |
| 369.350* | Chol | Cholesterol^c^ | [M-H2O+H]+ | 640.603 | unknown |  |  |
| 371.358 | Chol | Cholesterol derivative^LM,d^ | [M-H2O+H]+ | 645.561* | CE | CE(16:1)^a,b,c^ | [M+Na]+ |
| 383.333 | oxChol | Cholesterol derivative^LM,d^ |  | 647.577* | CE | CE(16:0)^a,b,c^ | [M+Na]+ |
| 385.348 | oxChol | Dehydrocholesterol^LM,d^ | [M+H]+ | 652.610 | unknown |  |  |
| 401.343* | oxChol | 7-ketocholestol^d^ | [M+H]+ | 654.628 | unknown |  |  |
| 428.370 | unknown |  |  | 656.630 | unknown |  |  |
| 429.375 | unknown |  |  | 666.495 | unknown |  |  |
| 430.379 | unknown |  |  | 668.525 | unknown |  |  |
| 431.385 | unknown |  |  | 668.622 | unknown |  |  |
| 496.346* | LPC | LPC(16:0)^d^ | [M+H]+ | 669.563 | CE | CE(18:3)^a^ | [M+Na]+ |
| 518.335* | LPC | LPC(18:3)^d^ | [M+H]+ | 671.580* | CE | CE(18:2)^a,b,c^ | [M+Na]+ |
| 520.351* | LPC | LPC(18:2)^d^ | [M+H]+ | 673.593* | CE | CE(18:1)^a,b,c^ | [M+Na]+ |
| 522.371* | LPC | LPC(18:1)^d^ | [M+H]+ | 675.573 | SM | SM(d32:1)^a,b^ | [M+H]+ |
| 524.374* | LPC | LPC(18:0)^d^ | [M+H]+ | 676.560 | unknown |  |  |
| 542.322* | LPC | LPC(20:5)^d^ | [M+H]+ | 678.623 | unknown |  |  |
| 544.340* | LPC | LPC(20:4)^d^ | [M+H]+ | 680.636 | unknown |  |  |
| 546.351* | LPC | LPC(20:3)^d^  LPC(18:0)^b^ | [M+H]+  [M+Na]+ | 681.643 | unknown |  |  |
| 549.488* | DG | DG(32:1)^a^ | [M-H2O+H]+ | 682.653 | unknown |  |  |
| 551.509 | DG | DG(32:0)^a^ | [M-H2O+H]+ | 683.658 | unknown |  |  |
| 575.510* | DG | DG(34:2)^a^  DG(O-32:1)^b^ | [M-H2O+H]+  [M+Na]+ | 685.567* | oxCE | Oxo-ODE-CE^d^ | [M+Na]+ |
| 577.520* | DG | DG(34:1)^a^  DG(O-32:0)^b^ | [M-H2O+H]+  [M+Na]+ | 687.566* | oxCE*  CE | HODE-CE^b^  CE(18:2)^a^ | [M+Na]+  [M+K]+ |
| 579.534 | DG | DG(34:0)^a^ | [M-H2O+H]+ | 689.570 | CE  TG | CE(18:1)^b^  TG(38:1)^b^ | [M+K]+  [M+Na]+ |
| 596.540 | unknown |  |  | 692.638 | unknown |  |  |
| 598.558 | unknown |  |  | 693.567* | CE | CE(20:5)^a,b^ | [M+Na]+ |
| 599.497* | DG | DG(36:4)^a^ | [M-H2O+H]+ | 695.581* | CE | CE(20:4)^a,b^ | [M+Na]+ |
| 601.512* | DG | DG(36:3)^a^  DG(O-34:2)^b^ | [M-H2O+H]+  [M+Na]+ | 695.659 | unknown |  |  |
| 603.530* | DG | DG(36:2)^a^  DG(O-34:1)^b^ | [M-H2O+H]+  [M+Na]+ | 696.673 | unknown |  |  |
| 605.555 | DG | DG(36:1)^a^ | [M-H2O+H]+ | 697.535* | SM | SM(32:1)^a,b^ | [M+Na]+ |
| 610.572 | unknown |  |  | 697.591* | CE | CE(20:3)^a,b^ | [M+Na]+ |
| 624.567 | unknown |  |  | 697.672 | unknown |  |  |
| 626.591 | unknown |  |  | 699.606 | CE | CE(20:2)^a,b^ | [M+Na]+ |
| 627.544 | DG | DG(38:4)^a^  DG(O-36:3)^b^ | [M-H2O+H]+  [M+Na]+ | 701.571 | SM | SM(34:2)^b^ | [M+H]+ |

*m/z = mass measured in MALDI-MSI experiment using Synapt G2Si TOF system. For exact mass measured with FTICR and ppm values, we refer to the online available METASPACE at https://metaspace2020.eu/ data named Human CEA Patient H - section 4 and Human CEA Patient I - section 4*

*lipid group = assigned lipid group based on database search, i.e. LipidMaps, HMDB.*

*ID = proven lipid identity, superscript denotes identification method: a. Lipidyzer MRM analysis, b. FTICR measurement combined with METASPACE database (FDR of maximum 10%), c. Identified in previous study(19), d. Identified from literature, LM: assigned from search in LipidMaps, HMDB: assigned from search in HMDB*

*adduct = positive ion adduct in identification experiment.*

** Asterisks denote the lipids that were included in the cross-correlation analysis*

^d^ *Lipid identity not confirmed in identification experiments due to methodological limitations. However m/z values have been identified as 7-ketocholesterol(14,61), other cholesterol derivatives(61), LPCs(62) and oxo-ODE-CE(12) in literature and have been included as such in our cross-correlation analysis.*

**Supplemental Table S3** – continued

| ***m/z ± 0.02*** | **lipid class** | **ID** | **adduct** | ***m/z ± 0.02*** | **lipid class** | **ID** | **adduct** |
| --- | --- | --- | --- | --- | --- | --- | --- |
| 703.583* | SM | SM(34:1)^a,b,c^ | [M+H]+ | 796.541 | PC | PC(34:2)^a,b^ | [M+K]+ |
| 705.589 | SM | SM(34:0)^b^ | [M+H]+ | 796.615 | PC-O | PC(O-38:4) ^LM,HMDB,b^ | [M+H]+ |
| 707.542 | unknown |  |  | 798.548 | PC | PC(34:1)^a,b^ | [M+K]+ |
| 711.550* | oxCE*  CE | CE(11:1D3)^HMDB^  CE(20:4)^a^ | [M+Na]+  [M+K]+ | 802.610 | unknown |  |  |
| 717.578 | unknown |  |  | 803.617 | unknown |  |  |
| 719.571* | CE | CE(22:6)^a,b^ | [M+Na]+ | 807.641* | SM | SM(40:2)^a,b^ | [M+Na]+ |
| 721.586* | CE | CE(22:5)^a,b,c^ | [M+Na]+ | 808.609 | PC | PC(36:2)^b^ | [M+Na]+ |
| 723.544 | SM | SM(34:2)^b^ | [M+Na]+ | 810.610* | PC | PC(38:4)^b,c^ | [M+H]+ |
| 725.565* | SM | SM(34:1)^a,b,c^ | [M+Na]+ | 811.676* | SM | SM(40:0)^b^ | [M+Na]+ |
| 727.566 | SM | SM(34:0)^b^ | [M+Na]+ | 813.695* | SM | SM(42:2)^a,b,c^ | [M+H]+ |
| 729.590* | SM | SM(36:2)^a,b^ | [M+H]+ | 815.710 | SM | SM(42:1)^a,b^ | [M+H]+ |
| 731.614* | SM | SM(36:1)^a,b,c^ | [M+H]+ | 816.597 | PC-O | PC(O-38:5) ^LM,HMDB,b^ | [M+Na]+ |
| 732.584 | unknown |  |  | 820.539* | PC | PC(36:4)^a^ | [M+K]+ |
| 733.561 | unknown |  |  | 822.555 | PC | PC(36:3)^a^ | [M+K]+ |
| 734.580* | PC | PC(32:0)^a,b,c^ | [M+H]+ | 823.592 | SM | SM(40:2)^a^ | [M+K]+ |
| 735.572 | unknown |  |  | 824.584 | unknown |  |  |
| 736.577 | unknown |  |  | 825.610 | SM | SM(40:1)^a^ | [M+K]+ |
| 739.550 | SM | SM(34:2)^LM,HMDB^ | [M+K]+ | 827.622 | unknown |  |  |
| 741.545* | SM | SM(34:1)^a,b^ | [M+K]+ | 827.709* | TG | TG(48:1)^a^ | [M+Na]+ |
| 743.555 | SM | SM(34:0)^LM,HMDB^ | [M+K]+ | 828.620 | unknown |  |  |
| 746.606 | PC-O | PC(O-34:1) ^LM,HMDB,b^ | [M+H]+ | 829.619 | unknown |  |  |
| 750.565 | unknown |  |  | 829.717* | TG | TG(48:0)^a^ | [M+Na]+ |
| 751.574 | SM | SM(36:2)^a,b^ | [M+Na]+ | 830.574 | PC | PC(40:8)^b^  PC(38:5)^b^ | [M+H]+  [M+Na]+ |
| 753.585 | SM | SM(36:1)^a,b^ | [M+Na]+ | 832.600 | PC | PC(38:4)^b^ | [M+Na]+ |
| 755.552 | unknown |  |  | 834.637 | unknown |  |  |
| 756.558* | PC | PC(32:0)^a,b^ | [M+Na]+ | 835.673* | SM | SM(44:5)^b^  SM(42:2) ^a,b^ | [M+H]+ [M+Na]+ |
| 758.574* | PC | PC(34:2)^a,c^ | [M+H]+ | 837.690 | SM | SM(42:1)^a,b^ | [M+Na]+ |
| 760.587* | PC | PC(34:1)^a,b,c^ | [M+H]+ | 839.590 | unknown |  |  |
| 762.618 | PC | PC(34:0)^HMDB,b^ | [M+H]+ | 846.661 | unknown |  |  |
| 764.652 | unknown |  |  | 847.593 | unknown |  |  |
| 766.578* | PC-O | PC(O-36:5) ^LM,HMDB,b^ | [M+H]+ | 848.588 | unknown |  |  |
| 766.754 | unknown |  |  | 848.676 | unknown |  |  |
| 768.581* | PC-O | PC(O-36:4) ^LM,HMDB,b^ | [M+H]+ | 849.611 | unknown |  |  |
| 772.542 | PC | PC(32:0)^a,b^ | [M+K]+ | 850.605 | unknown |  |  |
| 774.580 | unknown |  |  | 851.629 | SM | SM(42:2)^a,b^ | [M+K]+ |
| 776.584 | unknown |  |  | 853.721* | TG | TG(50:2)^a,b^ | [M+Na]+ |
| 780.564* | PC | PC(34:2)^a,b,c^ | [M+Na]+ | 855.592 | unknown |  |  |
| 782.577* | PC | PC(36:4)^a,c^  PC(34:1)^a,b^ | [M+H]+  [M+Na]+ | 855.737 | TG | TG(50:1) ^LM,HMDB,b^ | [M+Na]+ |
| 784.591* | PC | PC(36:3)^a,b,c^ | [M+H]+ | 856.596 | PC | PC(42:9)  PC(40:6)^HMDB^ | [M+H]+  [M+Na]+ |
| 786.610* | PC | PC(36:2)^a,b,c^ | [M+H]+ | 857.596 | unknown |  |  |
| 788.620* | PC | PC(36:1)^c^ | [M+H]+ | 857.750* | TG | TG(50:0)^a^ | [M+Na]+ |
| 790.585 | unknown |  |  | 860.774 | unknown |  |  |
| 792.600 | PC-O | PC(O-38:6) ^LM,HMDB,b^ | [M+H]+ | 862.790 | unknown |  |  |
| 794.614 | PC-O | PC(O-38:5) ^LM,HMDB,b^ | [M+H]+ | 870.684 | PC | PC(42:2) ^HMDB^ | [M+H]+ |

**Supplemental Table S3** – continued

| ***m/z ± 0.02*** | **lipid group** | **ID** | **adduct** |
| --- | --- | --- | --- |
| 872.693 | PC | PC(42:1) ^HMDB^ | [M+H]+ |
| 877.738* | TG | TG(52:4)^a,b^ | [M+Na]+ |
| 879.738* | TG | TG(52:3)^a,b^ | [M+Na]+ |
| 881.760* | TG | TG(52:2)^a,b^ | [M+Na]+ |
| 883.768* | TG | TG(52:1)^a,b^ | [M+Na]+ |
| 885.781* | TG | TG(52:0)^a^ | [M+Na]+ |
| 888.804 | unknown |  |  |
| 889.726 | unknown |  |  |
| 895.725 | unknown |  |  |
| 897.736 | PE-Cer | PE-Cer(t38:0)^b^ | [M+Na]+ |
| 901.569 | unknown |  |  |
| 901.732* | TG | TG(54:6)^a,b^ | [M+Na]+ |
| 903.751* | TG | TG(54:5)^a,b^ | [M+Na]+ |
| 905.751* | TG | TG(54:4)^a,b^ | [M+Na]+ |
| 907.773* | TG | TG(54:3)^a,b^ | [M+Na]+ |
| 909.794* | TG | TG(54:2)^a,b^ | [M+Na]+ |
| 923.750 | unknown |  |  |
| 927.744* | TG | TG(56:7)^a^ | [M+Na]+ |
| 929.768* | TG | TG(56:6)^a,b^ | [M+Na]+ |
| 931.778* | TG | TG(56:5)^a,b^ | [M+Na]+ |
| 932.570 | unknown |  |  |
| 933.790* | TG | TG(56:4)^a^ | [M+Na]+ |
| 940.770 | unknown |  |  |
| 955.774* | TG | TG(58:7)^a^ | [M+Na]+ |
| 956.570 | unknown |  |  |
| 958.583 | unknown |  |  |
| 980.570 | unknown |  |  |
| 984.602 | unknown |  |  |
| 986.769 | unknown |  |  |
| 1,010.774 | unknown |  |  |
| 1,012.783 | unknown |  |  |
| 1,014.798 | unknown |  |  |
| 1,034.772 | unknown |  |  |
| 1,036.783 | unknown |  |  |
| 1,038.805 | unknown |  |  |
| 1,040.817 | unknown |  |  |
| 1,042.824 | unknown |  |  |
| 1,077.583 | unknown |  |  |

**Supplemental Table S4.** Demographic information an histological tissue compositions per patient

| Patient | Age [yrs] | Sex | Intima area  (µ ± SD (max)) [mm^2^] | % Necrotic core  (µ ± SD) | % Foam cells  (µ ± SD) | % Fibrin  (µ ± SD) | % Erythrocytes  (µ ± SD) | Number of sections | Representative tissue section* |
| --- | --- | --- | --- | --- | --- | --- | --- | --- | --- |
| A | 56 | F | 19.0 ± 10.4 (34.4) | 10.1 ± 9.0 | 1.1 ± 1.7 | 0.9 ± 1.9 | 0.0 ± 0.1 | 9 |  |
| B | 69 | M | 22.2 ± 16.2 (54.5) | 14.4 ± 18.9 | 6.1 ±7.8 | 10.3 ± 17.4 | 0.6 ± 2.1 | 12 |  |
| C | 65 | M | 22.6 ± 6.9 (30.1) | 5.2 ± 4.8 | 5.1 ± 6.7 | 1.7 ± 3.5 | 0.0 ± 0.0 | 7 | 1 |
| D | 75 | M | 23.0 ± 13.9 (54.5) | 11.9 ± 17.6 | 4.6 ± 4.8 | 0.6 ± 1.1 | 3.7 ± 6.5 | 11 |  |
| E | 75 | M | 25.4 ± 11.4 (47.2) | 8.5 ± 9.4 | 2.9 ± 1.8 | 14.4 ± 13.6 | 0.0 ± 0.1 | 8 |  |
| F | 63 | M | 26.6 ± 11.6 (43.8) | 16.1 ± 11.0 | 3.1 ± 6.3 | 1.5 ± 2.3 | 0.3 ± 0.7 | 8 | 2 |
| G | 81 | M | 28.9 ± 17.2 (59.2) | 24.8 ± 17.1 | 0.6 ± 1.3 | 5.0 ± 5.9 | 0.0 ± 0.1 | 10 |  |
| H | 62 | F | 35.7 ± 11.1 (51.0) | 8.7 ± 3.1 | 0.3 ± 0.4 | 5.6 ± 4.4 | 10.2 ± 7.7 | 5 | 3 |
| I | 69 | M | 49.6 ± 16.4 (70.1) | 48.6 ± 23.8 | 3.0 ± 5.1 | 15.7 ± 14.2 | 0.0 ± 0.1 | 7 | 4 |
| J | 79 | M | 52.1 ± 28.5 (101.0) | 35.3 ± 17.5 | 0.6 ± 1.1 | 23.6 ± 22.9 | 0.0 ± 0.0 | 8 | 5 |
| K | 69 | M | 53.3 ± 18.3 (77.4) | 47.5 ± 21.3 | 0.9 ± 1.4 | 13.8 ± 14.5 | 0.3 ± 1.1 | 12 | 6 |
| L | 82 | M | 57.3 ± 37.9 (120.0) | 25.6 ± 21.4 | 0.4 ± 0.7 | 32.0 ± 31.3 | 0.8 ± 1.4 | 9 |  |

*% area of histological component relative to total intima area. µ = mean, SD = standard deviation.*

*µ ± SD: percentages were averaged over all tissue sections of a patient*

*µ ± SD intima area is given in mm^2^, areas were averaged over all tissue sections of a patient. Intima area of largest tissue section is reported between brackets*

** Representative tissue sections are referred to in Figure 1, 2 and 4.*

**Supplemental Figure S1.** Histological tissue composition of 12 carotid plaques


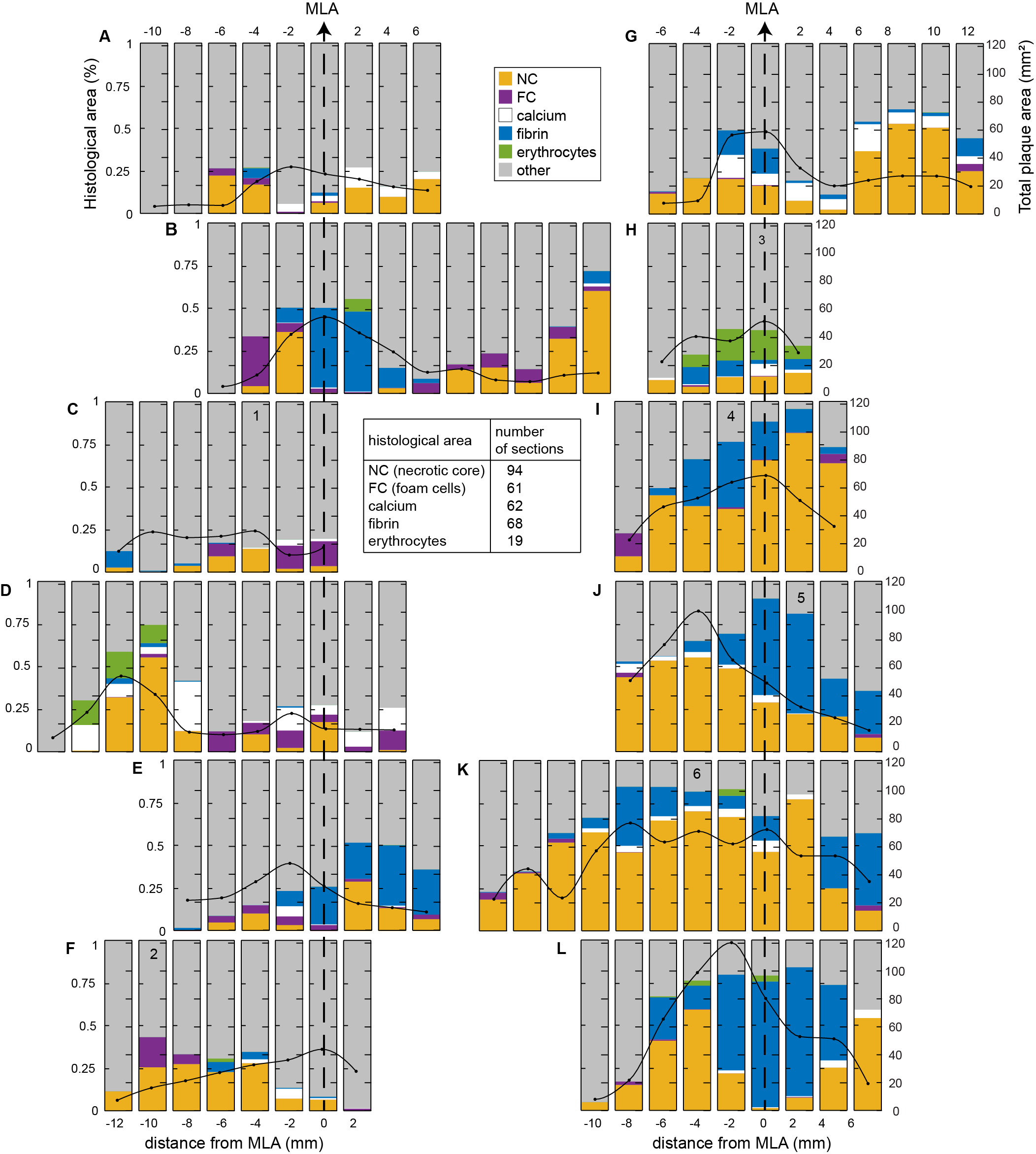


**Supplemental Figure S1**. Overview of plaque components present in each carotid endarterectomy (CEA) sample. A-L: Bar graphs show the relative proportions (%) of necrotic core (NC), foam cells (FC), calcifications, fibrin and erythrocytes compared to the total intima area, see left axes.Tissue labeled as other was not segmented, this part of the tissue was histologically heterogeneous, but did not fit into any accepted classifications of atherosclerotic tissues. Total intima area represents the area of the intima layer of the vessel. The black line superimposed on the bargraph shows the total intima area in mm^2^, see right axis. The numbers in the graphs correspond to the exemplatory sections shown in Figure 1, 2 and 4. The table in the middle shows the frequency of occurance of each histological component. MLA = minimal lumen area. Per CEA sample, the tissue section containing the minimal lumen area is denoted.

**Supplemental Figure S2.** MALDI-MSI of CE 18:1 compared to CE 18:2


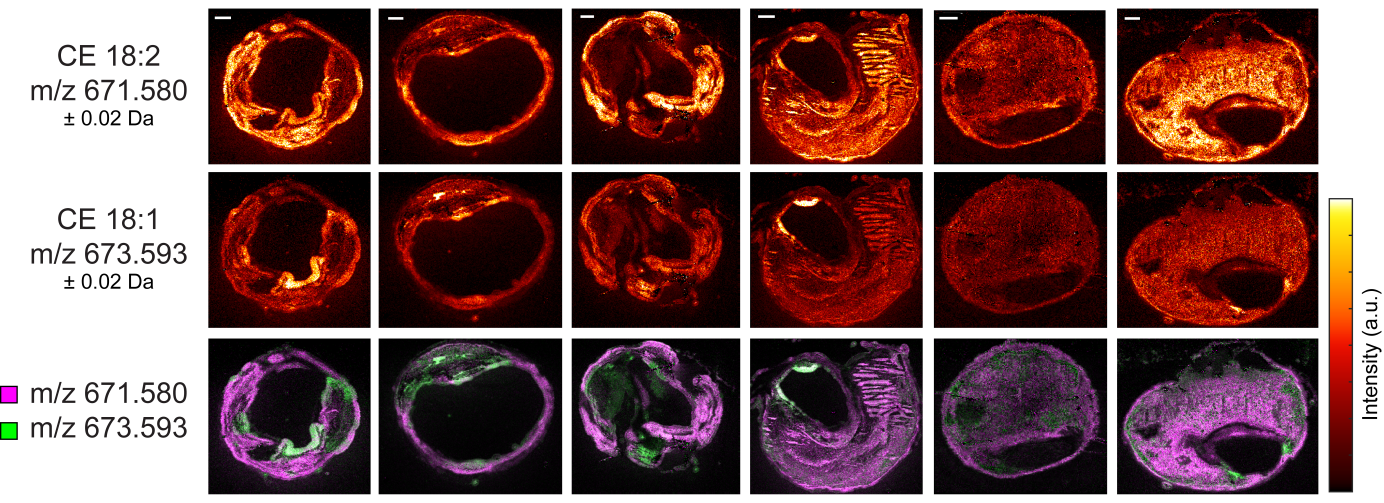
***Supplemental* *Figure S2.*** *MALDI-MS images on the same intensity scale of CE(18:2) with m/z 671.580 and CE(18:1) with m/z 673.593, and an overlay of both lipid images with CE(18:2) shown in purple and CE(18:1) shown in green.Sections are the same as depicted in Figure 1 and 4 of the manuscript.*

**Supplemental Figure** **S3**. MALDI-MSI of TG compared to DG
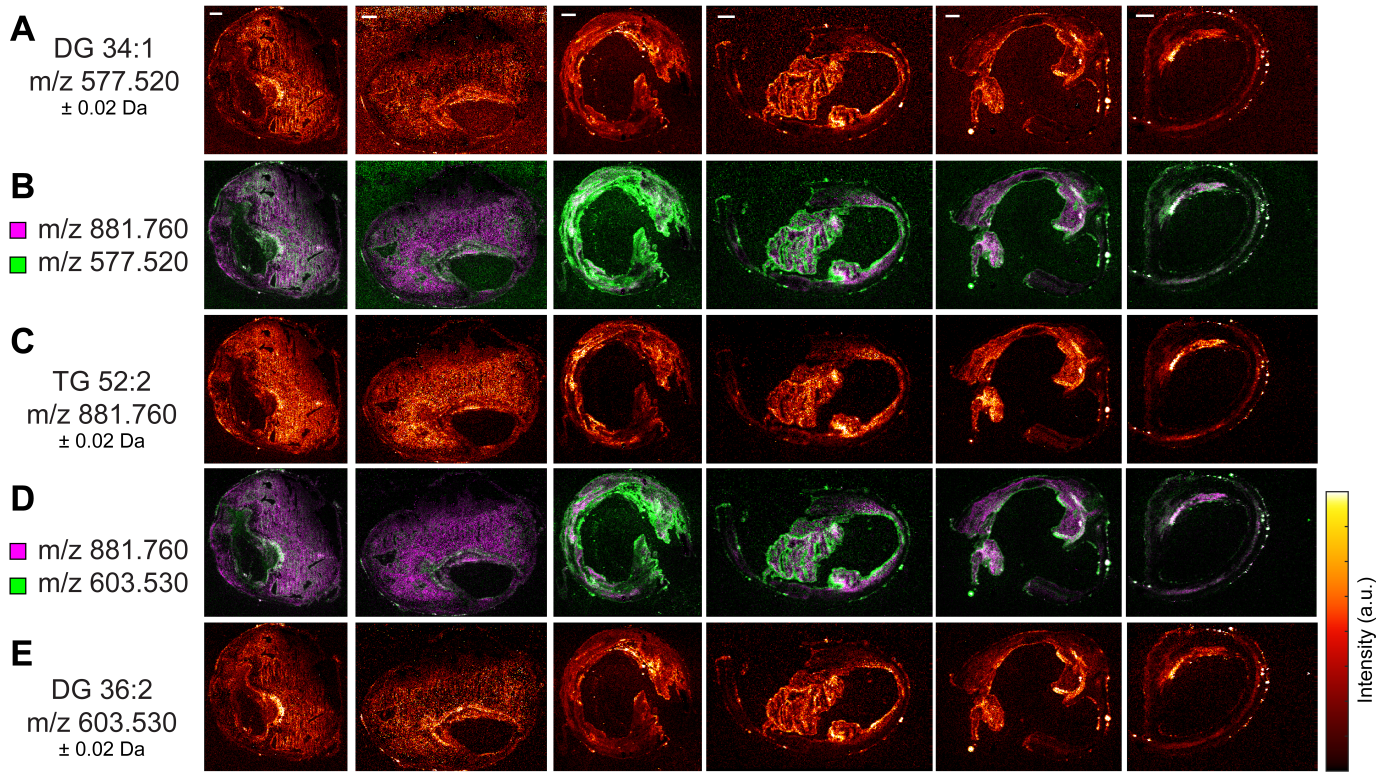


**Supplemental Figure S3.** MALDI-MS images of a) m/z 577.520, annotated as DG(34:1) and possibly DG(O-32:0), b) an overlay of lipid images with DG(34:1) shown in green and TG(52:2) shown in purple c) m/z 881.760 annotated as TG(52:2), d) an overlay of lipid images with DG (36:2) shown in green and TG(52:2) shown in purple and e) m/z 603.530 annotated as DG (36:2) and possibly DG(O-34:1)

**Supplemental Figure** **S4**. MALDI-MSI of PC compared to DG

*
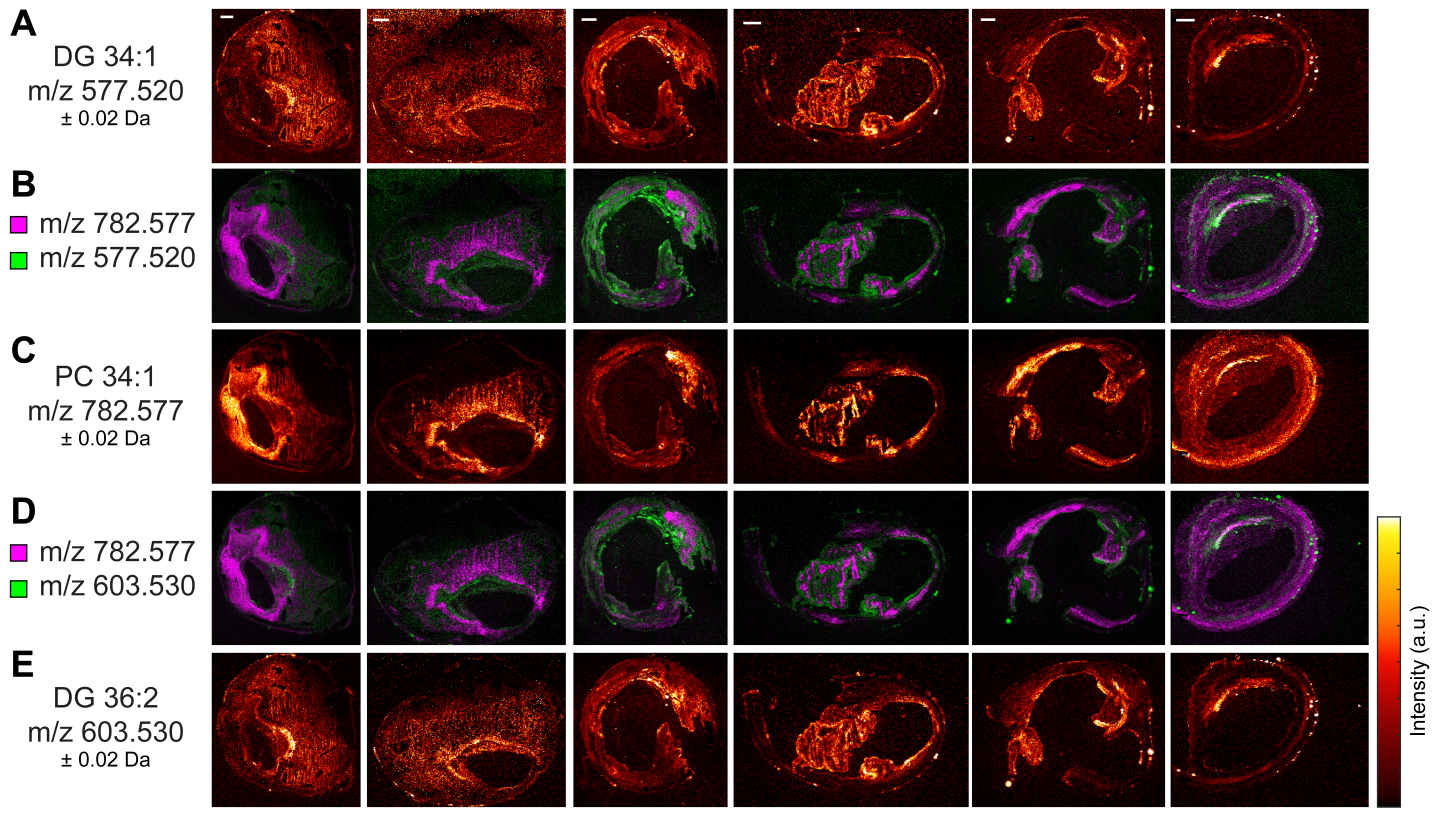
*

**Supplemental Figure S4.** MALDI-MS images of a) m/z 577.520, annotated as DG(34:1) and possibly DG(O-32:0), b) an overlay of lipid images with DG(34:1) shown in green and PC(34:1) shown in purple c) m/z 782.577 annotated as PC(34:1), d) an overlay of lipid images with DG (36:2) shown in green and PC(34:1) shown in purple and e) m/z 603.530 annotated as DG (36:2) and possibly DG(O-34:1). Sections are the same as depicted in Supplemental Figure S3.

**Supplemental Table S5.** OPLS-DA model parameters

| **Histological area** | **R^2^** | **Q^2^** | **CV-ANOVA** | **Patients included in model** *(number of patients in model / total number of patients)* ***** |
| --- | --- | --- | --- | --- |
| NC | 0.57 | 0.51 | 5.34E-19 | All except D, H, L *(9/12)* |
| Fibrin | 0.68 | 0.52 | 2.53E-07 | B, C, F, G, J, K *(6/12)* |
| Foam cells | 0.63 | 0.55 | 7.33E-15 | All except D *(11/12)* |
| Erythrocytes | 0.67 | 0.61 | 5.57E-05 | B, F, H, I, K, L *(6/12)* |

*This table summarizes the parameters for OPLS-DA models comparing the mean spectrum of a histological area (NC, fibrin, FCs or erythrocytes) to the mean spectrum of tissue outside this histological area. The patients included in the model are reported in the last column of this table, all tissue sections of included patients were added to the model. R^2^ and Q^2^ values represent the quality of fit and predictability of the model respectively. Significance of OPLS-DA models was checked by sevenfold cross-validation analysis of variance (CV-ANOVA).*

** The number of patients for which R^2^ and Q^2^ values were highest. Minimal 6 out of 12 patients were included in the model.*

**Supplemental Table S6.** Lists of *m/z* values with VIP>1.0 resulting from significant OPLS-DA models

**Necrotic core**

*m/z* values with VIP>1.0, resulting from the OPLS-DA model comparing NC with not-NC areas

| ***m/z* value** | **VIP** |  | ***m/z* value** | **VIP** |
| --- | --- | --- | --- | --- |
| 811.676 | 2.00 |  | 743.555 | 1.45 |
| 701.571 | 1.98 |  | 849.610 | 1.44 |
| 729.590 | 1.95 |  | 647.577 | 1.42 |
| 685.567 | 1.94 |  | 901.568 | 1.41 |
| 839.590 | 1.93 |  | 823.592 | 1.41 |
| 675.573 | 1.92 |  | 739.549 | 1.41 |
| 689.570 | 1.91 |  | 751.574 | 1.41 |
| 813.694 | 1.86 |  | 837.690 | 1.39 |
| 705.589 | 1.85 |  | 766.578 | 1.39 |
| 703.583 | 1.84 |  | 524.374 | 1.37 |
| 717.578 | 1.80 |  | 725.565 | 1.36 |
| 857.596 | 1.79 |  | 669.563 | 1.33 |
| 687.565 | 1.78 |  | 825.610 | 1.32 |
| 676.560 | 1.77 |  | 496.346 | 1.31 |
| 855.592 | 1.77 |  | 807.641 | 1.31 |
| 815.710 | 1.76 |  | 768.581 | 1.30 |
| 721.585 | 1.73 |  | 542.322 | 1.29 |
| 711.550 | 1.72 |  | 792.600 | 1.28 |
| 834.637 | 1.72 |  | 851.629 | 1.26 |
| 719.571 | 1.71 |  | 850.605 | 1.23 |
| 522.371 | 1.69 |  | 695.581 | 1.21 |
| 723.544 | 1.68 |  | 816.597 | 1.20 |
| 727.566 | 1.63 |  | 695.659 | 1.18 |
| 697.535 | 1.62 |  | 753.585 | 1.15 |
| 856.596 | 1.61 |  | 546.351 | 1.14 |
| 835.673 | 1.59 |  | 847.593 | 1.14 |
| 671.580 | 1.58 |  | 696.673 | 1.12 |
| 673.593 | 1.58 |  | 790.585 | 1.12 |
| 544.340 | 1.54 |  | 518.335 | 1.11 |
| 731.614 | 1.53 |  | 697.672 | 1.07 |
| 746.606 | 1.50 |  | 1,077.583 | 1.06 |
| 796.615 | 1.50 |  | 692.638 | 1.05 |
| 645.561 | 1.50 |  | 827.622 | 1.05 |
| 520.351 | 1.47 |  | 385.347 | 1.01 |
| 794.614 | 1.47 |  |  |  |

**Supplemental Table S6 – continued**

**Fibrin**

*m/z* values with VIP>1.0, resulting from the OPLS-DA model comparing fibrin with not-fibrin areas

| ***m/z* value** | **VIP** |  | ***m/z* value** | **VIP** |
| --- | --- | --- | --- | --- |
| 931.778 | 2.12 |  | 746.606 | 1.42 |
| 929.768 | 2.06 |  | 889.726 | 1.39 |
| 955.774 | 2.05 |  | 796.615 | 1.37 |
| 383.333 | 2.01 |  | 895.725 | 1.36 |
| 927.744 | 1.86 |  | 877.738 | 1.34 |
| 599.497 | 1.84 |  | 794.614 | 1.32 |
| 401.343 | 1.84 |  | 719.571 | 1.29 |
| 905.751 | 1.81 |  | 676.560 | 1.26 |
| 901.732 | 1.78 |  | 827.709 | 1.25 |
| 603.529 | 1.78 |  | 627.544 | 1.23 |
| 933.790 | 1.78 |  | 766.578 | 1.22 |
| 601.512 | 1.74 |  | 1,040.817 | 1.21 |
| 721.585 | 1.71 |  | 883.768 | 1.20 |
| 923.750 | 1.68 |  | 733.561 | 1.19 |
| 909.794 | 1.66 |  | 879.738 | 1.17 |
| 907.773 | 1.63 |  | 685.567 | 1.15 |
| 903.751 | 1.62 |  | 855.592 | 1.13 |
| 605.555 | 1.62 |  | 701.571 | 1.13 |
| 829.717 | 1.62 |  | 816.597 | 1.12 |
| 579.534 | 1.61 |  | 1,038.805 | 1.12 |
| 575.510 | 1.61 |  | 897.736 | 1.11 |
| 857.596 | 1.59 |  | 762.618 | 1.11 |
| 551.509 | 1.55 |  | 1,036.783 | 1.11 |
| 856.596 | 1.52 |  | 729.590 | 1.08 |
| 792.600 | 1.51 |  | 839.590 | 1.07 |
| 857.750 | 1.51 |  | 1,042.824 | 1.06 |
| 549.488 | 1.46 |  | 385.347 | 1.04 |
| 577.520 | 1.45 |  | 705.589 | 1.03 |
| 666.495 | 1.44 |  | 881.760 | 1.02 |
| 853.721 | 1.43 |  | 885.781 | 1.02 |
| 855.737 | 1.42 |  | 692.638 | 1.00 |

**Supplemental Table S6 – continued**

**Foam cells**

*m/z* values with VIP>1.0, resulting from the OPLS-DA model comparing foam cell with not-foam cell areas

| ***m/z* value** | **VIP** |  | ***m/z* value** | **VIP** |
| --- | --- | --- | --- | --- |
| 697.591 | 1.52 |  | 1,042.824 | 1.15 |
| 888.804 | 1.47 |  | 1,038.805 | 1.15 |
| 729.590 | 1.42 |  | 697.672 | 1.14 |
| 862.790 | 1.41 |  | 1,036.783 | 1.14 |
| 1,010.774 | 1.41 |  | 849.610 | 1.13 |
| 685.567 | 1.40 |  | 830.574 | 1.13 |
| 986.769 | 1.38 |  | 822.555 | 1.13 |
| 756.558 | 1.35 |  | 764.652 | 1.12 |
| 780.564 | 1.33 |  | 731.614 | 1.12 |
| 848.588 | 1.32 |  | 870.684 | 1.12 |
| 885.781 | 1.30 |  | 851.629 | 1.12 |
| 782.577 | 1.28 |  | 798.548 | 1.12 |
| 827.709 | 1.28 |  | 825.610 | 1.11 |
| 638.472 | 1.26 |  | 832.600 | 1.11 |
| 1012.78 | 1.26 |  | 673.593 | 1.11 |
| 784.591 | 1.26 |  | 1,034.772 | 1.10 |
| 732.584 | 1.24 |  | 735.572 | 1.10 |
| 850.605 | 1.24 |  | 796.541 | 1.07 |
| 829.717 | 1.24 |  | 827.622 | 1.07 |
| 755.552 | 1.22 |  | 810.610 | 1.05 |
| 808.609 | 1.22 |  | 733.561 | 1.05 |
| 860.774 | 1.22 |  | 701.571 | 1.05 |
| 758.574 | 1.22 |  | 1,014.798 | 1.04 |
| 734.580 | 1.21 |  | 855.592 | 1.04 |
| 772.542 | 1.18 |  | 839.590 | 1.04 |
| 627.544 | 1.17 |  | 932.570 | 1.04 |
| 824.584 | 1.17 |  | 676.560 | 1.02 |
| 848.676 | 1.16 |  | 846.661 | 1.01 |
| 786.610 | 1.16 |  | 687.565 | 1.00 |
| 760.587 | 1.15 |  |  |  |

**Supplemental Table S6 – continued**

**Erythrocytes**

*m/z* values with VIP>1.0, resulting from the OPLS-DA model comparing erythrocyte with not-erythrocyte areas

| ***m/z* value** | **VIP** |
| --- | --- |
| 932.570 | 1.35 |
| 780.564 | 1.35 |
| 782.577 | 1.34 |
| 958.583 | 1.33 |
| 756.558 | 1.32 |
| 758.574 | 1.32 |
| 786.610 | 1.31 |
| 784.591 | 1.31 |
| 768.581 | 1.31 |
| 760.587 | 1.30 |
| 832.600 | 1.29 |
| 956.569 | 1.29 |
| 810.610 | 1.29 |
| 790.585 | 1.28 |
| 734.580 | 1.28 |
| 796.541 | 1.28 |
| 830.574 | 1.26 |
| 808.609 | 1.26 |
| 772.542 | 1.25 |
| 735.572 | 1.24 |
| 798.548 | 1.24 |
| 820.539 | 1.22 |
| 984.602 | 1.21 |
| 822.555 | 1.21 |
| 788.620 | 1.20 |
| 905.751 | 1.19 |
| 980.570 | 1.17 |
| 766.578 | 1.15 |
| 736.577 | 1.14 |
| 903.751 | 1.11 |
| 923.750 | 1.11 |
| 762.618 | 1.11 |
| 707.542 | 1.10 |
| 796.615 | 1.08 |
| 897.736 | 1.07 |
| 816.597 | 1.07 |
| 929.768 | 1.07 |
| 755.552 | 1.05 |
| 877.738 | 1.03 |
| 931.778 | 1.03 |
| 955.774 | 1.02 |
| 652.609 | 1.01 |

**Supplemental Table S7.** Number of *m/z* values with VIP > 1.0 for the different multivariate models and the NMF component in which these *m/z* values are most abundant

| **NMF component** | **NC** | **Fibrin** | **FC** | **Erythrocytes** |
| --- | --- | --- | --- | --- |
| 1 | 18 | 4 | 9 | 0 |
| 2 | 2 | 4 | 0 | 0 |
| 3 | 0 | 21 | 2 | 10 |
| 4 | 2 | 0 | 0 | 0 |
| 5 | 39 | 13 | 1 | 0 |
| 6 | 22 | 9 | 21 | 29 |

**Supplemental references**

12. Hutchins, P. M., E. E. Moore, and R. C. Murphy. 2011. Electrospray MS/MS reveals extensive and nonspecific oxidation of cholesterol esters in human peripheral vascular lesions. J. Lipid Res. **52**: 2070–2083.

14. Malmberg, P., K. Börner, Y. Chen, P. Friberg, B. Hagenhoff, J.-E. Månsson, and H. Nygren. 2007. Localization of lipids in the aortic wall with imaging TOF-SIMS. Biochim. Biophys. Acta - Mol. Cell Biol. Lipids. **1771**: 185–195.

19. Visscher, M., A. M. Moerman, P. C. Burgers, H. M. M. Van Beusekom, T. M. Luider, H. J. M. Verhagen, A. F. W. Van der Steen, K. Van der Heiden, and G. Van Soest. 2019. Data Processing Pipeline for Lipid Profiling of Carotid Atherosclerotic Plaque with Mass Spectrometry Imaging. J. Am. Soc. Mass Spectrom. **30**: 1790–1800.

61. Hasegawa, M., H. Hakamata, I. Matsunaga, and F. Kusu. 2011. Detection of oxysterols in oxidatively modified low density lipoprotein by MALDI-TOF MS. Eur. J. Lipid Sci. Technol. **113**: 423–429.

62. Ravandi, A., S. Babaei, R. Leung, J. C. Monge, G. Hoppe, H. Hoff, H. Kamido, and A. Kuksis. 2004. Phospholipids and oxophospholipids in atherosclerotic plaques at different sTGes of plaque development. Lipids. **39**: 97–109.
